# Severe mental illness and infectious disease mortality: a systematic review and meta-analysis

**DOI:** 10.1016/j.eclinm.2024.102867

**Published:** 2024-10-09

**Authors:** Amy Ronaldson, Isabelle Nascimento Santana, Sophie Carlisle, Katie H. Atmore, Natasha Chilman, Margaret Heslin, Sarah Markham, Alex Dregan, Jayati Das-Munshi, Temi Lampejo, Matthew Hotopf, Ioannis Bakolis

**Affiliations:** a Health Service & Population Research Department, Institute of Psychiatry, Psychology, & Neuroscience (IoPPN), King's College London, UK; b East London NHS Foundation Trust, UK; c Department of Psychological Medicine, IoPPN, King's College London, UK; d Department of Biostatistics & Health Informatics, IoPPN, King's College London, UK; e South London and Maudsley NHS Foundation Trust, UK; f Infection Sciences, King's College Hospital NHS Foundation Trust, UK

**Keywords:** Severe mental illness, Schizophrenia, Bipolar disorder, Infection, Pneumonia, Mortality

## Abstract

**Background:**

Evidence from meta-analyses suggest that people with severe mental illness (SMI) are at increased risk of death from infectious diseases compared to those without SMI. However, few reviews have focused on mortality risk from specific infection types, apart from COVID-19. The aim of this systematic review and meta-analysis was to comprehensively describe and quantify the risk of death from infections (excluding COVID-19) in people with SMI, exploring specific infection types where possible.

**Methods:**

PubMed, Web of Science, PsycINFO, and EMBASE were searched for relevant studies up to 18th June 2024. Studies were included if they assessed the impact of SMI (bipolar disorder, schizophrenia and schizoaffective disorders, other psychoses) on risk of mortality from any infectious disease excluding COVID-19. Random-effects meta-analyses of the risk of death from ‘infectious diseases’, respiratory infections, sepsis, and ‘other’ infections in SMI were performed. The review protocol was registered in PROSPERO (CRD42023422151).

**Findings:**

Twenty-nine articles were included in the review. All were observational cohort studies carried out in high income countries and 59% were judged to be of good quality. Narrative analysis indicated that having SMI was associated with increased risk of death from infectious disease (23/29 studies), with mixed results for sepsis. People with SMI were more than twice as likely to die from ‘infectious diseases’ than the general population (pooled relative risk (RR) = 2.71, 95% confidence interval (CI) = 2.33–3.16, N = 739,852) and more than three times more likely to die from respiratory infections (pooled RR = 3.27, 95% CI = 2.57–4.17, N = 1,353,905). Sources of heterogeneity across studies included SMI diagnosis, gender, type of control group, and infection type.

**Interpretation:**

People with SMI are at an increased risk of death from infection, particularly from respiratory infections like influenza and pneumonia and should be prioritised for preventative strategies including influenza and pneumococcal vaccines. More work is needed to fully understand why infection mortality risk is increased in SMI.

**Funding:**

10.13039/100011705MQ Mental Health Research Fellowship MQF22∖12.

**Research in context**

**Evidence before this study**

Evidence from meta-analyses suggest that people with severe mental illness (SMI) are at increased risk of dying from infectious disease compared to the general population/people without SMI. However, few reviews have focused on the risk of death from specific types of infection, with the exception of COVID-19. Numerous reviews and meta-analyses have shown that people with SMI are at an increased risk of COVID-19 mortality. The risk factors that lead to this increased COVID-19 mortality are likely common factors that also contribute to increased risk of death from other types of infections, such as pneumonia, influenza, and sepsis. A search of electronic databases up to 18th June 2024 did not yield a published, comprehensive synthesis of evidence relating to infectious disease mortality in SMI. This review sought to fill this gap.

**Added value of this study**

Twenty-nine published papers were included in the review, and all comprised results from observational cohort studies from high-income countries. Meta-analysis showed that, compared to the general population or non-SMI controls, having SMI was associated with a doubling in the risk of dying from infectious disease. When we looked at different infection types, we found that people with SMI were more than three times more likely to die from respiratory infections. This was particularly pronounced for pneumonia (four-fold increase in pooled risk). Pooled risk of death from other types of infections, such as human immunodeficiency virus (HIV), hepatitis, and gastrointestinal infections, was also increased. More complex findings emerged for sepsis. We report no increased pooled risk of sepsis mortality, but subgroup analysis revealed that this was influenced by study design (i.e. type of control group).

**Implications of all the available evidence**

People with SMI are at increased risk of death from infectious diseases, particularly from respiratory infections like influenza and pneumonia. Although work is needed to unravel the factors involved, we would recommend that people with SMI are prioritised for preventative strategies including influenza and pneumococcal vaccines. Closer monitoring and medical follow-up in people with SMI diagnosed with certain infections might be warranted, particularly pneumonia. More generally, increasing public awareness about increased infectious disease mortality risk in this population will empower people with SMI to be vigilant about symptoms and when to seek medical attention.

## Introduction

Globally, people with severe mental illness (SMI) die 10–20 years earlier than the general population,^1^, ^2^, ^3^ and evidence suggests that this gap may be widening.^4^ Approximately 9% of this premature mortality is explained by unnatural causes such as suicide and self-harm.^3^ However, life expectancy in SMI is largely reduced because of physical health factors.

Several systematic reviews have described and quantified risk of death from non-communicable diseases in people with SMI, generally showing an increased risk of cardiovascular, respiratory, and cancer mortality.^2^^,^^5^^,^^6^ The evidence suggests that people with SMI are also at an increased risk of death from infectious diseases. Meta-analyses of mortality in people with schizophrenia^7^ and bipolar disorder^8^ have shown that people with these conditions are at increased risk of dying from infectious disease generally. Moreover, numerous reviews and meta-analyses have concluded that people with SMI are at increased risk of dying from COVID-19.^9^, ^10^, ^11^, ^12^, ^13^, ^14^, ^15^, ^16^ However, very few reviews have focused on mortality risk from other types of infection. There is some evidence that people with SMI are at in increased risk of death from pneumonia,^7^ and curiously at a decreased risk of mortality from sepsis and septic shock^17^ which requires further investigation.

There is need for a comprehensive review which focuses on studies looking at risk of death from infections (excluding COVID-19) in people with SMI. Therefore, in this paper we systematically reviewed the literature surrounding risk of infectious disease mortality in people with SMI, exploring specific types of infection where possible. Where appropriate, meta-analysis was used to quantify this risk.

## Methods

This review protocol is registered in the PROSPERO International Prospective Register of Systematic Reviews (CRD42023422151). The protocol conforms to the Preferred Reporting Items for Systematic Reviews and Meta-Analysis (PRISMA) guidelines (PRISMA checklist is provided in Supplementary Material). Ethical approval for this review was not required.

### Search strategy and selection criteria

We searched PUBMED, Web of Science, PsycINFO, and EMBASE from database origin to 13th June 2023 for relevant studies. An updated search was carried out on the 18th June 2024. Searches were carried out using both free text and, where possible, controlled vocabulary (e.g. MeSH terms). The search was supplemented with searches of grey literature^18^ and hand searches of relevant reference sections of relevant papers. Search strategies were formulated in line with the ‘PECO’ framework (Patient, Exposure, Comparator, Outcome).^19^ The full search strategy for each database is provided in Supplementary Material Tables S1a and b.

This review defined SMI as bipolar disorder, schizophrenia and schizoaffective disorder, and other psychoses as per guidance from National Health Service (NHS) England and the UK Quality and Outcomes Framework (QOF).^20^ We did not include major depressive disorder (MDD) in our definition of SMI but did extend our definition to include mood disorders with psychotic features (e.g. affective psychosis). Studies that included MDD in their definition of SMI were excluded unless results were presented separately for each SMI subtype (e.g. schizophrenia, bipolar disorder, affective psychosis).

Death from any infection was the primary outcome of the review. Studies were included if: 1) they were observational cohort studies, case–control studies, or randomised controlled trials; 2) estimates of mortality from any infection (excluding COVID-19) were provided in comparison with the general population or with a control group who did not have SMI (i.e. a statistical comparison was made between groups); 3) samples were not paediatric; 4) they were published in peer-reviewed journals. Studies were excluded if: 1) they were reviews, case reports, or studies that used qualitative methods only, book chapters were also excluded; 2) they were non-English-language articles; 3) they were conference proceedings; 4) the psychiatric disorder was acute (e.g., transient psychosis) or a result of an underlying medical condition (e.g., substance-induced psychosis, dementia-related psychosis).

### Study selection and extraction

Duplicate records were removed automatically using Rayyan.^21^ Articles were then independently screened in two stages: a title and abstract screen (AR, INS, SM), followed by the screening of potentially relevant full-text articles by several reviewers (AR, SC, KA, NC). Interrater reliability for the full-text screen was assessed using Cohen's kappa, which indicated substantial levels of interrater agreement between the reviewers (original search: κ 0.71, 89.3% agreement; updated search: κ 0.80, 93.1% agreement). Once selected for inclusion, the full texts of the articles were reviewed, and data were extracted by two reviewers (original search: AR, INS, updated search: AR, MH): AR extracted data from all publications and INS and MH extracted data from 50% of the studies in parallel and there was 86.3% agreement on extraction (updated extraction: 84.0% agreement). Conflicts at any stage of the selection and extraction process were resolved through discussion. Sample characteristics, methodological characteristics, and main infection mortality outcomes were extracted (Supplementary Table S2). The data extraction tables were piloted and refined before extraction began.

### Quality assessment

The Newcastle-Ottawa Scale (NOS)^22^ was used to assess the quality of all included studies due to their observational nature. The NOS assesses the quality of a study by awarding ‘stars’ based on three broad categories: selection of groups, comparability of groups, and discernment of the outcome of interest for the case–control or cohort. Each study is rated on nine factors and can earn a maximum of nine stars. A higher number of stars indicated less risk of bias in a study, and the number of stars determined whether a study is of good, fair, or poor quality. The quality of all studies was assessed by AR. MH assessed the quality of 50% of these studies in parallel and conflicts were resolved through discussion.

### Data analysis

A narrative synthesis was performed on all studies included in the review. Studies were grouped according to outcome: ‘infectious diseases’ (i.e. studies where infectious diseases were grouped together as a general category), respiratory infections, sepsis, and other infections. Meta-analysis was deemed appropriate if there were three or more separate studies on the same or similar outcome. The majority of studies reported standardised mortality ratios (SMRs), odds ratios (ORs), and hazard ratios (HRs). All effect statistics were treated as equivalent measures of risk to permit a comprehensive overview of associations.^23^ The term ‘relative risk (RR)’ was used throughout the review to describe results.

Effect statistics were pooled using inverse variance models, and were log transformed (natural logarithm) so that effect sizes were based on a normal distribution. The pooled log-effect statistics were then exponentiated for interpretation. In all meta-analyses, we used DerSimonian and Laird random-effects models which estimate pooled effects while considering heterogeneity between studies. Where studies ran more than one model, the results of the most adjusted model were entered into meta-analysis. Where studies reported crude mortality rates (e.g. observed deaths versus expected deaths), we calculated risk ratios and confidence intervals (CIs) manually.

Higgin's I^2^ was used to assess heterogeneity between studies in each meta-analysis. An I^2^ value greater than 50% is generally thought to indicate substantial heterogeneity and as a result statistical pooling is usually deemed inappropriate.^24^ However, this metric of heterogeneity may not be so reliable for meta-analyses containing a relatively small amount of studies.^25^ Therefore, we reported pooled effect statistics even where I^2^ values were high for ease of interpretation and due to the clinical relevance of results. These pooled estimates should be interpreted with caution.

Leave-one-out analysis was performed to identify potential outliers that may be overly influencing pooled effect sizes. Sources of heterogeneity were investigated using subgroup analyses. Sources investigated included SMI diagnosis type, sex, study quality, type of control group, as well as infection outcome (i.e. respiratory infection type) where appropriate. Metaregression was used to examine potential continuous sources of heterogeneity which included sample size, follow-up duration, and number of covariates adjusted for. Publication bias was assessed by visual examination of funnel plots and Egger linear regression tests.^26^ Note that in cases where study number was small (i.e. <3), we still performed subgroup analyses, metaregressions, and generated funnel plots for transparency.

All analyses were performed using STATA 18.0 (Stata Corp LLC, College Station, Texas).

### Role of the funding source

The funder of the study had no role in study design, data collection, data analysis, data interpretation, or writing of the report.

## Results

**Fig. 1:**
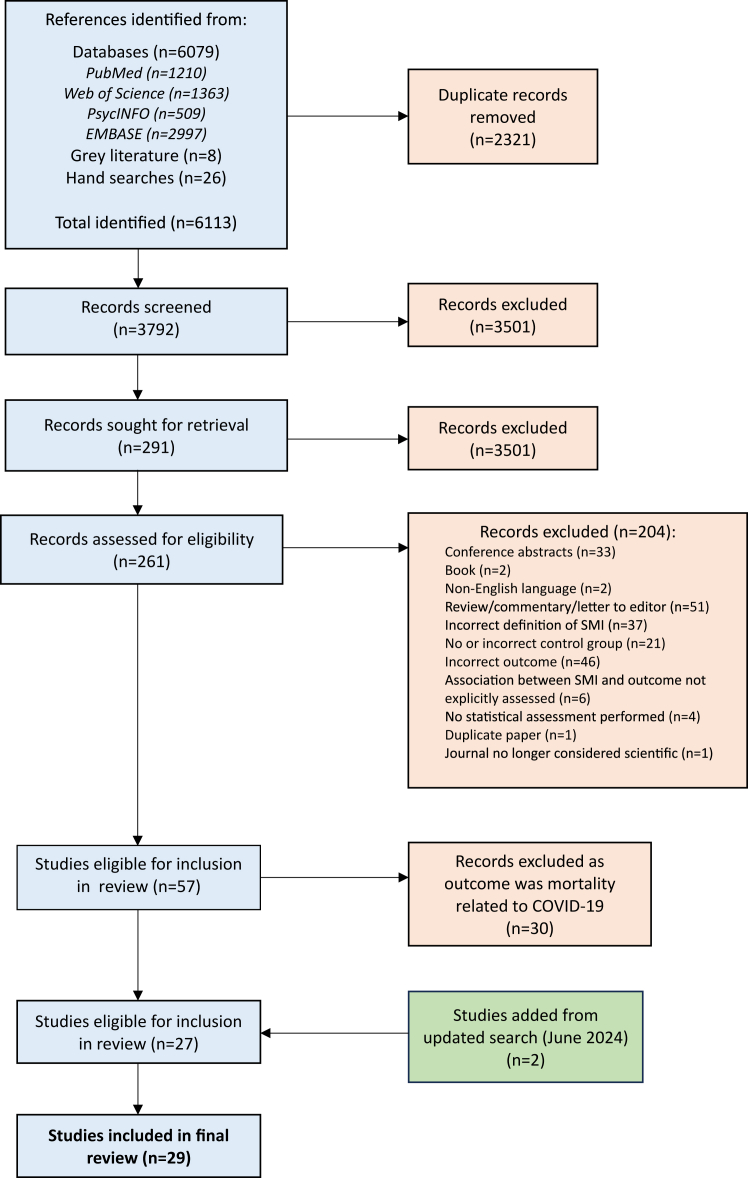
Preferred Reporting Items for Systematic reviews and Meta-Analyses (PRISMA) flow diagram.

The PRISMA flow diagram depicting the different phases of the systematic review is presented in Fig. 1 (updated search in Figure S1). The systematic literature search resulted in a total of 6079 records (updated search: 1449). Grey literature searches and hand searches of reference sections of relevant articles results in a further 34 records (updated search: 5 records). Following removal of duplicates, 3792 records remained (updated search: 932 records). After reviewing the titles and abstracts of these records, a total of 291 were eligible for review and sought for retrieval (updated search: 58 records). As we were unable to retrieve 30 articles (e.g. full text not accessible to staff performing the review), 261 were subjected to full-text screening. Of these, 57 were deemed suitable for inclusion in the review (updated search: 8 records). A list of excluded articles is provided in the Supplementary Material (Table S3a and d). Following removal of studies that assessed COVID-19 mortality (n = 30, updated search n = 6), 29 articles were included in the final review. All reported results from observational cohort studies. The majority of studies were of good quality (N = 17, 58.6%), 10 were of fair quality (34.5%), and two were of poor quality (6.9%). A detailed account of how the quality of each study was scored is provided in supplementary Table S4.

Details of the 29 included papers are provided in Table 1. All were from high income countries: Sweden (n = 6), United States (n = 5), Denmark (n = 4), United Kingdom (n = 3), Taiwan (n = 3), Canada (n = 2), Hong Kong (n = 2), Australia (n = 1), Spain (n = 1), Finland (n = 1), and France (n = 1). The number of participants with SMI ranged from 200 to 1.1 million and follow-up periods ranged from two to 40 years (with the exception of studies that were concerned with acute hospital outcomes). The majority of studies assessed infectious disease mortality in people with schizophrenia, schizoaffective disorder, and other psychoses (N = 23), nine studies included people with bipolar disorder, and three studies included SMI as a broader category. Fifteen studies examined mortality from ‘Infectious diseases’, 14 studies looked at death from Respiratory infections, six studies examined death from Sepsis, and four assessed mortality from Other infection types (e.g. gastrointestinal infection, pyelonephritis, human immunodeficiency virus (HIV)).

**Table 1:** Summary of included papers (N = 29).

Author, year	Country	Source of sample	SMI type (sample size)	Infection type(s)	Assessment of mortality	Study enrolment period	Follow-up duration	Comparison group	Outcome measure	Level of adjustment or standardisation	Result	Study quality
Allebeck & Wistedt, 1986	Sweden	Inpatient register	Schizophrenia (N = 1190)	Infections	Death register	1971	10 years	General population of the city	Standardised mortality ratio	Age and sex standardised	SMR = 4.4 (0.9–12.8)	Fair
Almeida et al., 2016	Australia	Community cohort study	Bipolar disorder (N = 256)	Infections; influenza or pneumonia	Death register	1996	13 years	Cohort participants free of bipolar disorder	Hazard ratio	Adjustment for age	*Infections:* HR = 1.35 (0.43–4.20) *Influenza or pneumonia:* HR = 3.66 (1.81–7.40)	Good
Brown et al., 2010	United Kingdom	Local patient register	Schizophrenia (N = 370)	Pneumonia	Death register	1981–1982	25 years	General population of the country	Standardised mortality ratio	Age and sex standardised	SMR = 835 (467–1377)	Good
Buda et al., 1988	United States	Inpatient register	Schizophrenia (N = 332)	Infections and parasitic diseases	Death certificates	1934–1945	30–40 years	General population of the state	Freeman-Tukey deviate	Age and sex standardised	Freeman-Tukey deviate ≥1.96, p < 0.01 Based on observed and expected mortality rates the author (AR) calculated SMR: SMR = 1.87 (0.99–2.75)	Fair
Castagnini et al., 2013	Denmark	National patient register	Bipolar disorder (N = 3200) Schizophrenia (N = 4576)	Infectious diseases	Death register	1995–2008	6.9 years	General population of the country	Standardised mortality ratio	Age and sex standardised	Bipolar disorder: No deaths Schizophrenia: SMR = 1.0 (0.1–7.2)	Good
Chan et al., 2021	Hong Kong	National patient register	Bipolar disorder (N = 12,556)	Infectious and parasitic diseases; non-aspiration pneumonia	Death register	2001–2016	Up to 11 years	General population of the country	Standardised mortality ratio	Age, sex, calendar period standardised	*Infectious and parasitic diseases:* SMR = 3.78 (2.70–5.04) *Non-aspiration pneumonia:* SMR = 4.72 (4.13–5.35)	Good
Chen et al., 2011	Taiwan	National patient register—patients hospitalised with pneumonia	Schizophrenia (N = 949)	Pneumonia—in-hospital death	Medical records	2002–2004	In hospital death	Pneumonia patients without schizophrenia matched on age, gender, year of admission, length of stay, Charlson Comorbidity Index	Odds ratio	Adjustment for age, gender, year of admission, length of stay, Charlson Comorbidity Index, physician characteristics, and hospital characteristics	OR = 1.60 (0.88–2.90)	Good
Cheng et al., 2014	Taiwan	Inpatient register	Schizophrenia (N = 2457)	Infectious and parasitic diseases	Death register	1995–1997	Up to 11 years	General population of the country	Standardised mortality ratio	Age and sex standardised	SMR = 4.7 (3.66–6.10)	Fair
Crump et al., 2013	Sweden	National patient register	Bipolar disorder (N = 6618)	Influenza or pneumonia	Death register	2001–2002	Up to 7 years	Population without bipolar disorder	Hazard ratio	Adjustment for age, marital status, education level, employment status, income, substance use disorders; stratified by sex	Women: HR = 3.52 (2.24–5.53) Men: HR = 3.85 (2.42–6.13)	Good
Crump et al., 2013	Sweden	National patient register	Schizophrenia (N = 8277)	Influenza or pneumonia	Death register	2001–2002	Up to 7 years	Population without schizophrenia	Hazard ratio	Adjustment for age, marital status, education level, employment status, income, substance use disorders; stratified by sex	Women: HR = 1.83 (1.63–2.06) Men: HR = 1.68 (1.52–1.86)	Good
Hiroeh et al., 2008	Denmark	National patient register	Schizophrenia (N = 54,595) Affective psychoses (N = 309,639) Nonaffective psychoses (N = 130,314)	Infectious diseases	Death Register	1973–1993	Up to 21 years	Population not admitted for psychiatric treatment	Standardised mortality ratio	Age and sex standardised	Schizophrenia: SMR = 103 (33–320) Affective psychoses: SMR = 141 (101–197) Nonaffective psychoses: SMR = 200 (122–236)	Fair
John et al., 2018	United Kingdom	National patient register	SMI (N = 27,979)	Septicaemia; Intestinal infections due to other specified organisms; Pneumonia	Death register	2004–2013	10 years	General population of the country	Standardised mortality ratio	Age and sex standardised	*Septicaemia*: SMR = 3.0 (2.0–4.1) *Intestinal infections due to other specified organisms:* SMR = 1.8 (1.0–3.0) *Pneumonia:* SMR = 3.8 (3.5–4.2)	Good
Kendler et al., 1986	United States	Twin register (male veterans)	Schizophrenia (N = 590)	Influenza or pneumonia	Death register	1917–1927	35 years	Twins in registry without schizophrenia	Standardised mortality ratio	Age and time-specific standardised	Based on SMR the author (AR) calculated associated 95% CI: SMR = 2.54 (1.21–3.87)	Fair
Ko et al., 2018	Taiwan	Local patient register	Schizophrenia (N = 4298)	Pneumonia	Death Register	1998–2010	Up to 13 years	General population of the country	Standardised mortality ratio	Age and sex standardised	SMR = 10.7 (4.5–17.9)	Fair
Lakbar et al., 2023	France	National patient register–patients hospitalised with septic shock	Schizophrenia (N = 3269) Bipolar disorder (N = 1923)	Septic shock	Medical records	2014–2018	90-day case fatality	Septic shock patients without SMI matched on age, sex, degree of social deprivation, year of hospitalisation	Hazard ratio	Adjustment for smoking, alcohol, and other substance addiction, overweight or obesity, Charlson comorbidity index, presence of trauma, surgical intervention, SAPS II score, organ failures, source of hospital admission, and time between hospital admission and ICU admission.	Schizophrenia: HR = 0.70 (0.65–0.75) Bipolar disorder: HR = 0.70 (0.63–0.76)	Good
Lesage et al., 2015	Canada	Provincial patient register	Schizophrenic disorders (women n = 14,242; men N = 19,418)	Infectious diseases	Death register	1999–2012	Up to 11 years	General population of the province	Standardised mortality ratio	Age standardised; stratified by sex	Women: SMR = 2.14 (1.43–3.20) Men: SMR = 3.89 (2.78–5.45)	Good
Mortensen et al., 1990	Denmark	National inpatient register	Schizophrenia (N = 6152)	Pyelonephritis	Death registry	1957	29 years	General population of the country	Standardised mortality ratio	Age and sex standardised	SMR = 1.47 (1.14–1.87)	Fair
Nilsson et al., 2021	Sweden	National patient register	Any psychotic disorder or bipolar disorder (N = 97,034)	Influenza or pneumonia; Sepsis	Death registry	2017	2 years	Population without any psychotic disorder or bipolar disorder	Odds ratio	None	*Influenza or pneumonia:* OR = 2.06 (1.87–2.27) *Sepsis:* OR = 1.61 (1.38–1.89)	Poor
Olaya et al., 2023	Catalonia, Spain	National patient register	Schizophrenia (N = 34,289) Other non-organic psychoses (N = 21,589) Bipolar disorder (N = 13,808)	Infectious diseases	Death register	2005	12 years	Age, sex, and geographically matched population from the general population	Hazard ratio	Adjustment for mental comorbidity	Schizophrenia: HR = 2.62 (1.83–3.76) Other non-organic psychoses: HR = 4.4 (2.65–7.29) Bipolar disorder: HR = 2.42 (1.20–4.86)	Good
Olfson et al., 2015	United States	National patient registry	Schizophrenia (N = 1,138,853)	Influenza or pneumonia; Sepsis	Death register	2001–2007	7 years	General population of the country	Standardised mortality ratio	Age, sex, race/ethnic group standardised	*Influenza or pneumonia:* SMR = 7.0 (6.7–7.4) *Sepsis:* SMR = 4.6 (4.3–4.8)	Good
Osby et al., 2000	Sweden	National inpatient register	Schizophrenia (N = 7784)	Infectious diseases	Death register	1973–1995	Up to 23 years	General population of the city	Standardised mortality ratio	Age, time of follow-up standardised; stratified by sex	Men: SMR = 3.4 (1.4–7.1) Women: SMR = 1.9 (0.6–4.3)	Fair
Osby et al., 2001	Sweden	National inpatient register	Unipolar disorder^b^ (N = 39,182) Bipolar disorder (N = 15,386)	Infectious diseases	Death register	1973–1995	Up to 23 years	General population of the country	Standardised mortality ratio	Age, time of follow-up standardised; stratified by sex	*Unipolar disorder* Men: SMR = 2.5 (1.7–3.5) Women: SMR = 2.0 (1.4–2.8) *Bipolar disorder* Men: SMR = 3.4 (1.9–5.8) Women: SMR = 2.4 (1.2–4.1)	Fair
Oud & Garza, 2022	United States	State patient register–patients hospitalised with sepsis	Schizophrenia and other psychotic disorders (N = 358)	Sepsis in-hospital mortality	Medical records	2014–2017	In hospital death	People hospitalised with sepsis with no mental disorder	Odds ratio	Adjustment for age, gender, race/ethnicity, primary health insurance, Deyo comorbidity index, congestive heart failure, chronic lung disease, cerebrovascular disease, diabetes, chronic renal disease, liver disease, malignancy, alcohol use disorders, and substance use disorders	OR = 0.74 (0.69–0.79)	Good
Ranger et al., 2023	United Kingdom	National patient register—people hospitalised with severe acute respiratory infections	Psychotic disorders (diagnosis and treatment) (N = 3255) Schizophrenia (N = 4587) Bipolar disorder (N = 3121)	Severe acute respiratory infections (influenza and pneumonia; Other acute lower respiratory infections)	Death register	2015–2020	Up to 5 years	People hospitalised with severe acute respiratory infections with no psychotic disorder, schizophrenia, bipolar disorder	Hazard ratio	Adjustment for age, sex, BMI, ethnicity, Townsend index of socio-economic deprivation, smoking and alcohol consumption, comorbidities, other medications	*Psychotic disorders:* HR = 2.91 (2.75–3.08) *Schizophrenia:* HR = 2.56 (2.40–2.73) *Bipolar disorder:* HR = 2.07 (1.92–2.24)	Good
Ribe et al., 2015	Denmark	National patient register	SMI (N = 11,343) Schizophrenia (N = 7388) Bipolar disorder (N = 3955)	Any infectious cause of death within 30 days of hospital admission for infection (any infection, sepsis, pneumonia, other respiratory, gastrointestinal, urinary tract, central nervous system, HIV and hepatitis, other infections)	Death register	1995–2011	Up to 17 years	People hospitalised for infection without SMI	Mortality rate ratio	Age, sex, calendar period standardised	SMI: MRR = 2.61 (2.30–2.96) Schizophrenia: MRR = 1.71 (1.58–1.85) Bipolar disorder: MRR = 1.27 (1.15–1.40) [Note: schizophrenia and bipolar disorder were excluded from meta-analyses to avoid including duplicate samples]	Good
Talaslahti et al., 2012	Finland	National inpatient register	Schizophrenia or schizoaffective disorder (N = 9461)	Infectious diseases	Death register	1969–1998	Up to 10 years	General population of the country	Standardised mortality ratio	Age and sex standardised	SMR = 26.6 (20.0–34.6)	Fair
Tsuang et al., 1980	United States	Inpatient register	Schizophrenia (N = 200)	Infective diseases	Death certificates; interviews; medical records	1934–1944	Up to 40 years	General population of the state	Observed -expected deaths	Age and sex standardised	Men: O - E = 1.77 (NS) Women: O-E = 3.80, p < 0.01^a^ Based on observed and expected mortality rates the author (AR) calculated SMR: Men: SMR = 8.69 (−3.39 to 20.79) Women: SMR = 20 (0.4–39.6)	Poor
Yazdani et al., 2022	Canada	Provincial patient register	Schizophrenia (N = 1079)	HIV-related mortality	Death register	1996–2017	Up to 21 years	People with HIV and no psychotic disorder	Hazard ratio	Adjustment for gender, age at cohort entry, lifetime diagnosis of hepatitis C co-infection, interaction term between substance use disorder and depression, suppressed HIV viral load	HR = 0.85 (0.66–1.69)	Good
Yung et al., 2020	Hong Kong	National patient register	Schizophrenia or schizoaffective disorder (N = 46,896)	Infectious and parasitic diseases; Non-aspiration pneumonia	Death register	2001–2016	Up to 11 years	General population of the country	Standardised mortality ratio	Age, sex, calendar period standardised	*Infectious and parasitic diseases:* SMR = 2.83 (2.44–3.26) *Non-aspiration pneumonia:* SMR = 4.60 (4.37–4.85)	Good

BMI, body mass index; E, expected; HR, hazard ratio; HIV, human immunodeficiency virus; ICU, intensive care unit; MRR, mortality rate ratio; NS, non-significant; O, observed; OR, odds ratio; SAPS II, Simplified Acute Physiology Score II; SMI, severe mental illness; SMR, standardised mortality ratio.

Meta-analyses notes: In some cases SMRs were divided by 100 before entry to meta-analysis (Brown et al. (2010), Hiroeh et al. (2008)).

a Tsuang et al. (1980) report results for 0–9 years, 10–29 year, and 30+ year follow-up periods. We report results from closest to 10 years of follow-up (i.e. 0–9 years).

b Osby et al. (2001) define unipolar disorder as different types of affective psychoses (ICD-8 296.00, 296.20, 296.88, 296.99, 298.00 and ICD-9 296B, 296D, 296X, 298A).

### SMI and risk of death from ‘infectious diseases’

The majority of studies (12/15, 80.0%) found that SMI was associated with increased mortality from ‘infectious diseases’.^27^, ^28^, ^29^, ^30^, ^31^, ^32^, ^33^, ^34^, ^35^, ^36^, ^37^, ^38^ The remaining studies reported no significantly increased mortality risk.^39^, ^40^, ^41^ Some studies suggested possible sex differences in the strength of associations^33^^,^^34^^,^^37^ and differences in risk across SMI diagnoses.^30^

**Fig. 2:**
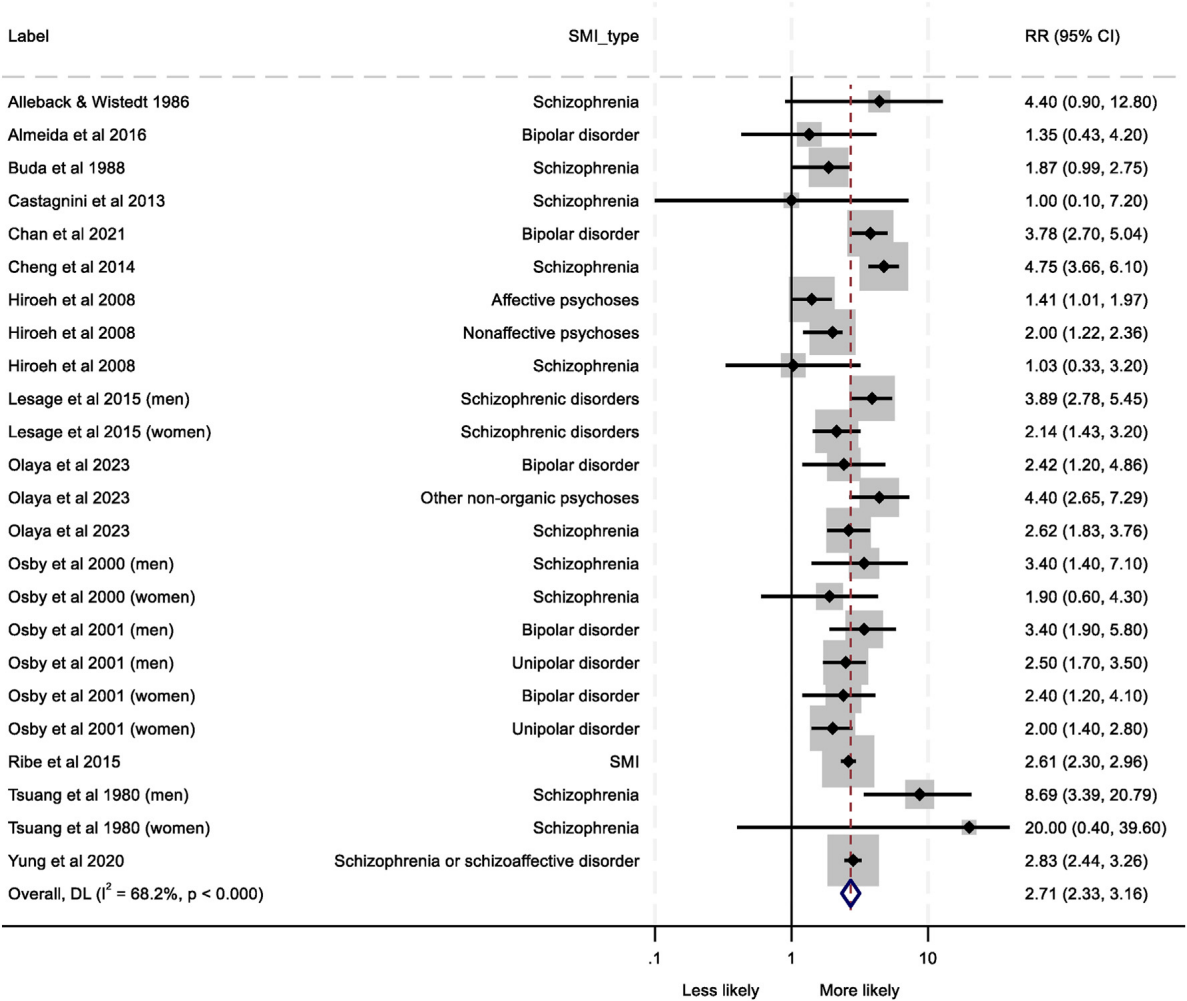
Forest plot depicting the pooled relative risk (RR) of death from ‘infectious diseases’. Severe mental illness (SMI) type for each analysis, relative risk and 95% confidence intervals (CIs), percentage heterogeneity (I^2^) and p values from χ2 tests of heterogeneity are displayed. Note: Osby et al. (2001) defined unipolar disorder as different types of affective psychoses (ICD-8 296.00, 296.20, 296.88, 296.99, 298.00 and ICD-9 296B, 296D, 296X, 298A).

Twenty-five analyses from 15 studies were eligible for meta-analysis.^27^, ^28^, ^29^, ^30^, ^31^, ^32^, ^33^, ^34^, ^35^, ^36^, ^37^, ^38^, ^39^, ^40^, ^41^ Leave-one-out analysis revealed that one study^36^ may have been overly influencing the pooled effect size and was removed from the meta-analysis (see Figure S2), leaving 24 analyses from 14 studies (N = 739,852 approximately as one study^37^ did not provide a SMI sample size). Seven studies were of good quality, six studies were of fair quality, and one was poor. Heterogeneity between studies was substantial (I^2^ = 68.2%). The pooled RR (Fig. 2) showed that patients with SMI were more than twice as likely to die from ‘infectious diseases’ than the general population or non-SMI control groups (pooled RR = 2.71, 95% CI = 2.33–3.16).

Subgroup analyses are provided in Table S5 and Figures S5. Gender (p = 0.044) emerged as a significant source of heterogeneity. Men showed the highest risk of death from ‘infectious diseases’ overall (pooled RR = 3.55, 95% CI = 2.58–4.89) relative to women (pooled RR = 2.14, 95% CI = 1.70–2.71) and combined samples (pooled RR = 2.61, 95% CI = 2.14–3.18). Metaregression (Table S9) revealed that follow-up period was related to the pooled effect size in that higher follow-up periods were associated with increased likelihood of death from ‘infectious diseases’ (β = 0.87, p < 0.003). Visual inspection of the funnel plot (Figure S6) indicated no publication bias (Egger's test p = 0.936).

### SMI and risk of death from respiratory infections

Thirteen of 14 studies reported increased likelihood of death from respiratory infection in people with SMI.^3^^,^^28^^,^^35^^,^^38^^,^^40^^,^^42^, ^43^, ^44^, ^45^, ^46^, ^47^, ^48^, ^49^ The remaining study reported no evidence of increased mortality risk.^50^ All studies looked at risk of death from pneumonia and/or influenza.

**Fig. 3:**
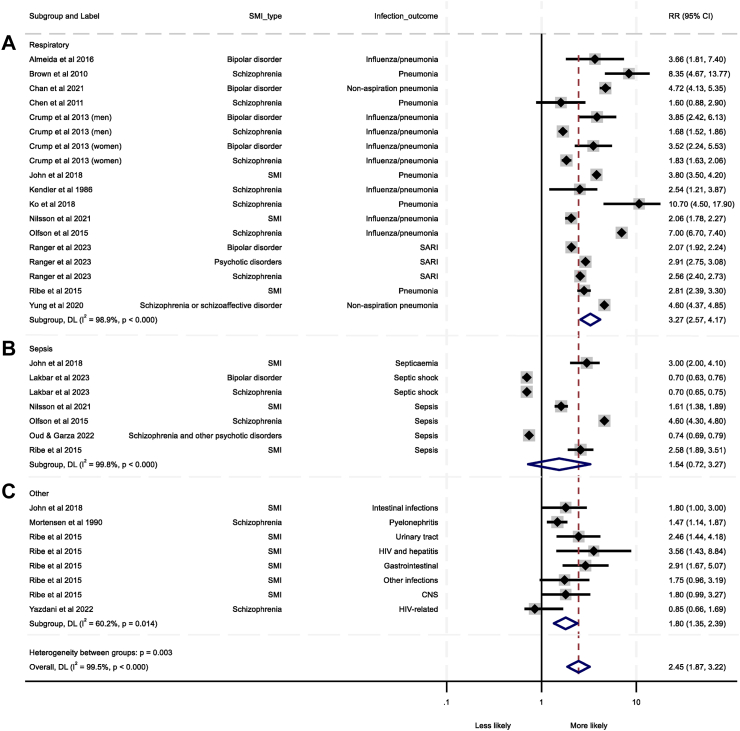
Forest plots of the pooled relative risk (RR) data for death from (A) Respiratory infections, (B) Sepsis, and (C) Other infection types. Severe mental illness (SMI) type for each analysis, relative risk and 95% confidence intervals (CIs), percentage heterogeneity (I^2^) and p values from χ2 tests of heterogeneity are displayed. CND, Central nervous system; SARI, Severe acute respiratory infection; SMI, Severe mental illness.

Eighteen analyses from 14 studies were eligible for meta-analysis (N = 1,353,905).^3^^,^^28^^,^^35^^,^^38^^,^^40^^,^^42^, ^43^, ^44^, ^45^, ^46^, ^47^, ^48^, ^49^, ^50^ Leave-one-out analysis revealed no outliers (Figure S3). Eleven studies were of good quality, two were fair, and one was poor. Heterogeneity between studies was high (I^2^ = 98.9%). Patients with SMI were more than three times more likely to die from respiratory infections than the general population or non-SMI controls (pooled RR = 3.27, 95% CI = 2.57–4.17, Fig. 3).

Regarding heterogeneity (Table S6), type of respiratory infection emerged as borderline significant (p = 0.051, Figure S7) with a stronger pooled effect size seen for pneumonia (pooled RR = 4.15, 95% CI = 3.42–5.04) compared to studies that grouped influenza and pneumonia together (pooled RR = 2.78, 95% CI = 1.95–3.96). Type of control group used was also a significant source of heterogeneity (p = 0.024, Figure S8) where a stronger pooled effect size was seen for studies that compared people with SMI with the general population (pooled RR = 3.87, 95% CI = 2.76–5.42) relative to studies that compared people with SMI with non-SMI patient groups or cohorts (pooled RR = 2.53, 95% CI = 2.19–2.93). Metaregression revealed no significant sources of heterogeneity (Table S9). There was also no evidence of publication bias (Funnel plot: Figure S9, Egger's test p = 0.389).

### SMI and risk of death from sepsis

Six studies assessed the risk of death from sepsis in people with SMI. Four studies reported significantly increased risk,^3^^,^^35^^,^^47^^,^^48^ whereas two studies reported significantly decreased risk.^51^^,^^52^

Seven analyses from six studies were eligible for meta-analysis (N = 1,282,577).^3^^,^^35^^,^^47^^,^^48^^,^^51^^,^^52^ Leave-one-out analysis revealed no outliers (Figure S4). Five of the studies were good quality, and one was poor. Heterogeneity between studies was high (I^2^ = 99.8%). The pooled RR indicated that people with SMI were at no greater risk of dying from sepsis than the general population/non-SMI controls (pooled RR = 1.54, 95% CI = 0.72–3.27, Fig. 3).

SMI diagnosis type (p < 0.001) and type of control group (p = 0.006) emerged as potential sources of heterogeneity (Table S7). Those with SMI were more likely to die from sepsis (pooled RR = 2.27, 95% CI = 1.50–3.43, Figure S10), but associations for schizophrenia/other psychoses and bipolar disorder remained non-significant. Interestingly, when studies comparing those with SMI to the general population were analysed separately, having SMI was associated with an increased risk of sepsis mortality (pooled RR = 2.81, 95% CI = 1.29–6.16, Figure S11), but the association remained non-significant for pooled studies where inpatient groups were used as controls. Metaregression revealed that higher sample sizes (β = 0.97, p = 0.049) and greater covariate adjustment (β = 0.88, p = 0.013) were significantly associated with increased likelihood of death from sepsis in people with SMI (Table S9). Funnel plots (Figure S12) and Egger's test (p = 0.789) indicated no publication bias was present.

### SMI and risk of death from other infections

Four studies assessed the risk of death from other infections, which included gastrointestinal infections,^3^^,^^35^ HIV and hepatitis-related mortality,^35^^,^^53^ pyelonephritis,^54^ as well as central nervous system, urinary tract, and other infections.^35^ All studies reported increased risk of death from infections in people with SMI, with the exception of John et al. (2018) who examined risk of death from intestinal infections and reported no increased risk.^3^

Eight analyses from four studies (N = 93,743) were entered into a meta-analysis (I^2^ = 60.2% indicating substantial heterogeneity).^3^^,^^35^^,^^53^^,^^54^ Three studies were good, and one was fair. Leave-one-out analysis identified no outliers (Figure S5). The pooled effect size indicated that people with SMI were significantly more likely to die from other infections than the general population/non-SMI controls (pooled RR = 1.80, 95% CI = 1.35–2.39, Fig. 3). SMI type emerged as a source of heterogeneity (Table S8) with studies looking at people with SMI reporting an increased pooled likelihood of dying (pooled RR = 2.20, 95% CI = 1.73–2.81, Figure S13), but studies looking at people with schizophrenia reporting no increased pooled risk (pooled RR = 1.16, 95% CI = 0.68–1.98). Metaregression (Table S9) revealed that the number of covariates adjusted for was associated with increased likelihood of death from other infection types (β = 0.89, p = 0.029). A funnel plot (Figure S14) and Egger's test (p = 0.201) indicate that publication bias was likely not an issue.

### Lived experience commentary

This systematic review and meta-analysis describing and quantifying the risk of death from infectious diseases (excluding COVID-19) in people with SMI has yielded unsurprising but clinically relevant results. Unsurprising in that the finding that people with SMI are at an increased risk of death from infectious diseases (particularly from respiratory infections like influenza and pneumonia) makes intuitive sense in the context of the long-acknowledged inequalities in mortality, higher smoking rates and obesity, and clinically relevant as this highlights the need for people with SMI to be prioritised for preventative strategies like pneumococcal and influenza vaccines. However, it is surprising given the high relevance of mortality inequalities in people with SMI that there was a paucity in the involvement of people with lived experience of SMI in the included studies. Lived experience involvement in such studies would have provided potentially invaluable sense-checking and dissemination advice for such studies. In addition to highlighting a clear need for more research to be done to elucidate the mechanisms which lead to people with SMI being at an increased risk of death from infectious diseases in order for better preventative interventions to be deployed, this systematic review and meta-analysis emphasises the need for people with SMI to be better epistemically and motivationally empowered to adopt self-protective measures such as smoking cessation, weight management and increased engagement in physical activity, and for changes in policy, commissioning and practice to support this.

## Discussion

The evidence from this systematic review and meta-analysis indicates that people with SMI are at an increased risk of dying from infectious diseases. Specifically, meta-analysis showed that people with SMI are more than twice as likely to die from ‘infectious diseases’ than the general population or non-SMI controls. Moreover, people with SMI are more than three times as likely to die from non-COVID respiratory infections, with this risk being especially pronounced for pneumonia (more than four-fold). This is particularly salient as lower respiratory infections are considered to be responsible for the most communicable disease deaths globally, and are the fourth leading cause of death amongst all disease categories.^55^ We also found that people with SMI were more likely to die from other types of infections (e.g. HIV, hepatitis, gastrointestinal infections). Interestingly, meta-analysis revealed that people with SMI were at no increased risk of death from sepsis, but this appeared to be influenced by study design.

The World Health Organisation (WHO) have suggested that people with SMI are at a 4- to 8-fold increased risk of death from infection.^56^ The results of the current meta-analyses, reporting an approximate 2- to 4-fold risk of death from infection in this population, partially support this and are also in line with previous meta-analyses which have reported 3-fold and 4-fold risk of mortality from infectious diseases in people with schizophrenia^7^ and bipolar disorder^8^ respectively. This discrepancy between the level of risk reported by the WHO and those reported by recent meta-analyses might be explained by the absence of studies from low- and middle-income countries (LMICs) qualifying for inclusion in reviews. Inclusion of data from LMICs, where infectious diseases such as malaria, tuberculosis, and HIV/AIDS are among the leading causes of death,^55^ might lead to stronger pooled effect sizes more in line with the risk reported by WHO. Nevertheless, with data from high-income countries we have shown that risk of death from pneumonia is increased more than 4-fold in people with SMI. This pooled effect size is larger than those reported in meta-analyses of COVID-19 mortality, where people with SMI have been found to have approximately double the odds of death compared to people without SMI.^13^^,^^14^

A recent meta-analysis of four studies reported a *decreased* risk of sepsis mortality in people with SMI.^17^ The results of the current meta-analysis are not in alignment with this previous review, as we found no clear evidence for increased, or decreased, pooled risk of death from sepsis. It is possible these contrasting conclusions might be explained by the inclusion of studies with differing study designs. Lakbar and authors^17^ included mostly studies with inpatient samples and inpatient control groups. The current review included six studies—three of which had population control groups,^3^^,^^47^^,^^48^ and three which had inpatient control groups.^35^^,^^51^^,^^52^ Subgroup analysis in the current study confirmed the importance of type of control showing that studies with population control groups had significantly increased pooled risk of death from sepsis, but studies with inpatient control groups had no pooled increased risk. It is entirely possible that while those with SMI do worse when compared with the general population, something more complex is going on when comparisons are made with hospitalised sepsis patients with some evidence suggesting people with SMI might be at a clinical advantage. Further investigation is needed to unpick sepsis mortality risk in SMI.

The elevated risk of death from infection in people with SMI is likely due to several interrelated mechanisms. First, patients with SMI experience significant health inequalities, reduced access to healthcare, poor quality service provision and mental-illness stigma. For example, people with SMI may experience ‘diagnostic overshadowing’ where symptoms of a physical disease are assumed to be a manifestation of the mental illness.^57^ Moreover, experience of stigma in healthcare settings might dissuade helpseeking.^58^ Second, SMI-related factors such as SMI diagnosis and severity might contribute to infectious disease mortality. Previous research has shown that the risk of infectious disease mortality is more pronounced in people with schizophrenia (as opposed to bipolar disorder).^35^^,^^49^ Severity of SMI symptoms might contribute to reduced uptake of preventive care and delayed presentation to healthcare which could lead to increased infection mortality risk, but more work is needed to understand how specific SMI symptoms (e.g. delusions, paranoia, social withdrawal) are involved.

Third, comorbidities commonly seen in SMI (e.g. diabetes, chronic respiratory diseases^59^) might exacerbate infectious disease mortality risk.^60^^,^^61^ Moreover, health behaviours such as smoking as well as alcohol and substance use are more prevalent in those with SMI^62^ and are known to be associated with worse infection outcomes.^63^, ^64^, ^65^, ^66^ Higher smoking prevalences and higher rates of respiratory disease (e.g. chronic bronchitis, chronic obstructive pulmonary disorder (COPD)) seen in SMI^67^ might explain the particularly strong pooled effect size for pneumonia mortality risk reported in the current meta-analysis.

Fourth, some antipsychotics are known to lead to weight gain, hyperlipidaemia, and diabetes, which in turn are associated with poor infectious disease outcomes.^68^ Conversely, efficacious antipsychotic medication regimes might protect against risk of mortality through an improvement in functioning, mental state, and ability to self-care.^69^ There is evidence that there may be clinically relevant interactions between various atypical antipsychotics and a range of antimicrobial agents which could lead to reduced drug efficacy and/or increased toxicity impacting control of infection, SMI, or both.^70^ Uncertainty around these interactions might lead to prescribing hesitancy, delayed or altered treatment, and poorer outcomes. For example, one of the most important interventions known to reduce mortality in sepsis is early appropriate antimicrobial administration and each additional hour of delay further increases risk of death.^71^ The role of antipsychotic medication in infection mortality risk is complex and in need of further investigation.

Finally, immune abnormalities have been shown to be a feature in severe mental disorders.^72^ SMI is characterised by several systemic immune-inflammatory changes which likely have implications for immunity from infection and ability to recover.^73^ For example, people with schizophrenia and bipolar disorder have been found to have increased levels of pro-inflammatory cytokines and acute phase proteins,^74^^,^^75^ as well as abnormal lymphocyte subpopulation counts.^76^ Further evidence for immune compromise in SMI comes from vaccine challenge studies, some of which show attenuated antibody responses to vaccines in this population.^77^ It is plausible that these immune abnormalities seen in SMI will have negative consequences for infectious disease trajectories. More work is needed to understand how all these potentially relevant sociodemographic, behavioural, clinical, and biological factors might interact to increase infection mortality risk in people with SMI. Since data on these factors are not usually reported in mortality studies, this meta-analysis could not explore how they contribute to mortality risk.

A considerable strength of this review is that all studies (at title and abstract, and full-text stage) were double screened by reviewers. The majority of studies included in this review were of good quality with large sample sizes, and in most cases used medical record linkage and established diagnostic codes to define SMI as well as infectious disease outcomes. However, a considerable number of studies were rated as fair or poor quality, largely owing to only including those who had received inpatient care for SMI in study samples. This compromised generalisability of these studies considering only a small proportion of people with severe mental health disorders receive inpatient care.^78^ Nevertheless, study quality did not emerge as a significant source of heterogeneity between studies included in meta-analyses. Although all efforts were made to exclude studies that included acute and transient psychotic disorders in their definition of SMI, it is possible that diagnostic codes relating to these types of conditions were included in broader definitions and were not picked up in screening. Although there was no explicit evidence of publication bias, the inclusion of only peer-reviewed publications meant some degree of publication bias was likely. Moreover, for the meta-analyses relating to sepsis and other infections the study sample was likely too low to be able to assess publication bias effectively. Despite using comprehensive literature search strategies across several databases, it is possible that retrieval of all relevant research was not complete. The exclusion of non-English-language studies might also have been a source of bias. However, there is evidence to suggest that excluding non-English studies from systematic reviews does not significantly impact results of meta-analyses.^79^

I^2^ values indicated significant heterogeneity between studies in all meta-analyses. This is not surprising in a review of this kind where there will be variation between studies in terms of SMI diagnosis, SMI sample size, study design, as well as infectious disease outcome, among other factors. Despite heterogeneity, we decided to pool effect statistics for ease of interpretability and due to the clinical relevance of the results. Examining sources of variance revealed that certain pooled effects differed across SMI type, gender, and type of control group. However, other factors likely explain the bulk of heterogeneity and the results of the meta-analyses presented in this review should be interpreted with this in mind. Metaregression also revealed that sample size, length of follow-up period, and number of covariates also contributed to heterogeneity in some of the meta-analyses. The results of the subgroup analyses and metaregressions should be interpreted with caution as sometimes the number of studies included was too small to be able to draw meaningful conclusions. One significant limitation of this review is that only studies from high-income countries qualified for inclusion in the review meaning that these results are not generalisable to LMICs where infectious diseases contribute far more to mortality rates.

Although more work is needed to unravel the factors involved, it is clear from the evidence presented in this review that people with SMI are at an increased risk of death from infection, and in particular pneumonia and influenza. Based on a two-fold risk of COVID-19 mortality, people with SMI were prioritised for COVID-19 vaccination in several countries.^80^ In this meta-analysis, we report a greater than three-fold increase in likelihood of death from influenza and pneumonia and would therefore recommend people with SMI are prioritised for preventative strategies including influenza and pneumococcal vaccines. We would also suggest consideration for the inclusion of people with SMI amongst the clinical groups deemed to be at increased risk of severe influenza for whom antiviral treatment and post-exposure prophylaxis may be recommended. Closer medical follow-up and monitoring in people with SMI diagnosed with certain infections might be warranted, particularly pneumonia. More generally, increasing public awareness about increased infectious disease mortality risk in this population will empower people with SMI to be vigilant about symptoms and when to seek medical attention.

In conclusion, the evidence from this systematic review and meta-analysis suggests that people with SMI are at increased risk of mortality from infectious disease, and respiratory infections in particular. Based on these results, we would recommend that this population are prioritised for preventative strategies including influenza and pneumococcal vaccines. More work is needed to fully understand why infection mortality risk is increased in SMI, and why results for sepsis are more mixed. Further efforts should be made to study this association in LMICs where infections are among the leading causes of death.

## Contributors

AR, AD, IB, JDM, and TL conceived the study, AR developed the research protocol and all data collection instruments and led data collection and analysis. AR wrote the first draft of the manuscript with input from JDM, TL, and SM. INS and SM helped with title and abstract screening as well as data extraction. SC, KA, and NC helped with full-text screening. MH assisted with the quality assessment of included studies. AR, INS, and MH accessed and verified the data. All authors contributed to the drafting and revision of the manuscript and had full access to all data in the study and had final responsibility for the decision to submit for publication. AR acquired funding for this project.

## Data sharing statement

Data collected for the study (i.e. extracted data), analytic code, or any other materials used in the review can be made available with publication by contacting the corresponding author: amy.ronaldson@kcl.ac.uk.

## Declaration of interests

MH declares funding from the National Institute for Health and Care Research (NIHR) and participates on the Equally Safe at School Trial Steering Committee. All other authors declare no competing interests.
