# Supplementary material for: Severe mental illness and infectious disease mortality: a systematic review and meta-analysis
Source: eClinicalMedicine. 2024 Oct 9;77:102867. doi: 10.1016/j.eclinm.2024.102867 (PMC11625019; doi:10.1016/j.eclinm.2024.102867)
Supplement: Supplementary Tables [file mmc1.docx]

**Severe mental illness and infectious disease mortality: A systematic review and meta-analysis**

**Supplementary Material**

**Contents**

[1. Search Strategy and Results 2](#_Toc174534821)

[2. List of data items which were extracted from each paper included in the review 8](#_Toc174534822)

[3. PRISMA flow diagram for updated search (June 2024) 9](#_Toc174534823)

[4. List of excluded studies 10](#_Toc174534824)

[5. Study quality assessment 27](#_Toc174534825)

[6. Leave-one-out analyses 30](#_Toc174534826)

[7. Subgroup analyses: Infectious disease overall 33](#_Toc174534827)

[8. Funnel plot: Infectious disease overall 35](#_Toc174534828)

[9. Subgroup analyses: Respiratory infections 36](#_Toc174534829)

[10. Funnel plot: Respiratory infections 41](#_Toc174534830)

[11. Subgroup analyses: Sepsis 42](#_Toc174534831)

[12. Funnel plot: Sepsis mortality 45](#_Toc174534832)

[13. Subgroup analyses: Other Infections 46](#_Toc174534833)

[14. Funnel plot: Other infection mortality 48](#_Toc174534834)

[15. Meta-regressions for all outcomes 49](#_Toc174534835)

[Supplementary Materials: References 50](#_Toc174534836)

1. Search Strategy and Results

The search strategies were designed based on the ‘PECO’ framework – Population, Exposure, Comparator, Outcome. Only studies with a comparison group were included in the review and therefore this element was deemed not suitable for inclusion in the search strategies.

Please note that ‘COVID-19’ was included in search terms so that the number of papers focusing on this particular infectious disease outcome could be quantified.

| **Table S1a.** Search strategies (searches performed between 7-12 June 2023) | | | | | |
| --- | --- | --- | --- | --- | --- |
| **PubMed (All Field Vocabulary)** | | | | | |
| # | | Searches | | | Results |
| 1  Population | | "Severe mental illness" [All Fields] OR "Serious mental illness" [All Fields] OR "SMI" [All Fields] OR Bipolar [All Fields] OR "Manic depression" [All Fields] OR Psychosis [All Fields] OR Psychoses [All Fields] OR Schizophrenia [All Fields] OR Schizoaffective [All Fields] | | | **307536** |
| 2  Exposure | | Infection [All Fields] OR Infectious [All Fields] OR "Communicable disease" [All Fields] OR Virus [All Fields] OR Viral [All Fields] OR Bacteria* [All Fields] OR Fungus [All Fields] OR Fungal [All Fields] OR Parasit* [All Fields] OR Pneumonia [All Fields] OR Influenza [All Fields] OR COVID-19 [All Fields] OR Tuberculosis [All Fields] OR Sepsis [All Fields] OR [All Fields] Septicaemia OR [All Fields] Septicemia [All Fields] OR Hepatitis [All Fields] OR HIV [All Fields] | | | **6395930** |
| 3  Outcome | | Death [All Fields] OR Mortality [All Fields] OR Life expectancy [All Fields] OR Survival [All Fields] | | | **3353715** |
| 4 | | #1 AND #2 AND #3 | | | **1677** |
| Additional filters | | ‘Human only’ | | | **1267** |
| Additional filters | | ‘English language only’ | | | **1137** |
| **PubMed (Controlled Vocabulary ‘MeSH terms’)** | | | | | |
| # | | Searches | Results | | |
| 1  Population | | Schizophrenia and disorders with psychotic features [MeSH Terms] OR Bipolar disorders [MeSH Terms] OR Affective psychoses [MeSH Terms] OR Disorder, psychotic [MeSH Terms] | **195578** | | |
| 2  Exposure | | Infectious disease [MeSH Terms] OR Viral disease [MeSH Terms] OR Virus [MeSH Terms] OR Bacteria [MeSH Terms] OR Parasitic disease [MeSH Terms] OR Pneumonia [MeSH Terms] OR influenza virus [MeSH Terms] OR tuberculosis [MeSH Terms] OR sepsis [MeSH Terms] OR septicemia [MeSH Terms] OR hepatitis a virus [MeSH Terms] OR hepatitis b virus [MeSH Terms] OR hepatitis c virus [MeSH Terms] OR hiv infection [MeSH Terms] | **3761148** | | |
| 3  Outcome | | Life expectancy [MeSH Terms] OR Mortality [MeSH Terms] OR Analysis, survival [MeSH Terms] | **732658** | | |
| 4 | | #1 And #2 AND #3 | **82** | | |
| Additional filters | | ‘Human only’ | **80** | | |
| Additional filters | | ‘English language only’ | **73** | | |
| **Web of Science (All Field Vocabulary ‘Topic’)**  Note: Web of Science Core Collection employs no controlled vocabulary | | | | | |
| # | | Searches | | | Results |
| 1  Population | | Topic: "Severe mental illness" OR Topic: Serious mental illness OR Topic: SMI OR Topic: Bipolar OR Topic: Manic depression OR Topic: Psychosis OR Topic: Psychoses OR Topic: Schizophrenia OR Topic: Schizoaffective | | | **388959** |
| 2  Exposure | | Topic: Infection OR Topic: Infectious OR Topic: Communicable disease OR Topic: Virus OR Topic: Viral OR Topic: Bacteria* OR Topic: Fungus OR Topic: Fungal OR Topic: Parasit* OR Topic: Pneumonia OR Topic: Influenza OR Topic: COVID-19 OR Topic: Tuberculosis OR Topic: Sepsis OR Topic: Septicaemia OR Topic: Septicemia OR Topic: Hepatitis OR Topic: HIV | | | **4863543** |
| 3  Outcome | | Topic: Death OR Topic: Mortality OR Topic: "Life expectancy" OR Topic: Survival | | | **3405297** |
| 4 | | #1 And #2 AND #3 | | | **1420** |
| Additional filters | | ‘English language only’ | | | **1363** |
| **PsycINFO (All Field Vocabulary)** | | | | | |
| # | | Searches | | | Results |
| 1  Population | | Severe mental illness OR Serious mental illness OR SMI OR Bipolar OR Manic depression OR Psychosis OR Psychoses OR Schizophrenia OR  Schizoaffective | | | **229769** |
| 2  Exposure | | Infection OR Infectious OR Communicable disease OR Virus OR Viral OR Bacteria OR Bacterial OR Fungus OR Fungal OR Parasite OR Parasitic OR Pneumonia OR Influenza OR COVID-19 OR Tuberculosis OR Sepsis OR Septicaemia OR Septicemia OR Hepatitis OR HIV | | | **137657** |
| 3  Outcome | | Death OR Mortality OR Life expectancy OR Survival | | | **186669** |
| 4 | | #1 And #2 And #3 | | | **461** |
| **PsycINFO (Controlled Vocabulary ‘Subject heading search’)** | | | | | |
| # | | Searches | | Results | |
| 1  Population | | Serious mental illness/ OR bipolar disorder/ or exp bipolar i disorder/ or exp bipolar ii disorder/ or exp mania/ or exp affective psychosis/ OR  psychosis/ or affective psychosis/ or brief psychotic disorder/ or capgras syndrome/ or chronic psychosis/ or delusional disorder/ or paranoid psychosis/ or reactive psychosis/ or schizophrenia/ or paranoid schizophrenia/ OR schizophrenia/ or schizoaffective disorder/ | | **156739** | |
| 2  Exposure | | infectious disorders/ or bacterial infections/ or parasitic infections/ or pneumonia/ or sepsis/ or sexually transmitted diseases/ or viral infections/ OR pneumonia/ or coronavirus/ or covid-19/ OR influenza/ or swine influenza/ OR tuberculosis OR sepsis/ OR hepatitis/ OR hiv/ or aids/ | | **88612** | |
| 3  Outcome | | "death and dying"/ or mortality rate/ or mortality risk/ OR life expectancy/ | | **46574** | |
| 4 | | #1 And #2 And #3 | | **47** | |
| **EMBASE (All Field Vocabulary)** | | | | | |
| # | | Searches | | Results | |
| 1  Population | | Severe mental illness.tw OR Serious mental illness.tw OR SMI.tw OR Bipolar.tw OR Manic depression.tw OR Psychosis.tw OR Psychoses.tw OR Schizophrenia.tw OR Schizoaffective.tw | | **336351** | |
| 2  Exposure | | Infection.tw OR Infectious.tw OR Communicable disease.tw OR Virus.tw OR Viral.tw OR Bacteria*.tw OR Fungus.tw OR Fungal.tw OR Parasit*.tw OR Pneumonia.tw OR Influenza.tw OR COVID-19.tw OR Tuberculosis.tw OR Sepsis.tw OR Septicaemia.tw OR Septicemia.tw OR Hepatitis.tw OR HIV.tw | | **4818250** | |
| 3  Outcome | | Death.tw OR Mortality.tw OR Life expectancy.tw OR Survival.tw | | **3902168** | |
| 4 | | #1 AND #2 AND #3 | | **1758** | |
| **EMBASE (Structured Vocabulary)** | | | | | |
| # | Searches | | | Results | |
| 1  Population | bipolar disorder/ or mania/ or bipolar depression/ or bipolar i disorder/ or bipolar ii disorder/ or bipolar mania/ OR psychosis/ or affective psychosis/ or schizophrenia/ OR schizophrenia/ or treatment-resistant schizophrenia/ | | | **357164** | |
| 2  Exposure | infection/ or virus infection/ OR communicable disease/ OR pneumonia/ OR influenza/ or swine influenza/ OR coronavirus disease 2019/ OR tuberculosis/ OR sepsis/ or septicemia/ OR virus hepatitis/ or hepatitis a/ or hepatitis b/ or hepatitis c/ OR Human immunodeficiency virus/ | | | **1874103** | |
| 3  Outcome | mortality/ or all-cause mortality/ or infection fatality ratio/ or mortality rate/ or premature mortality/ or standardized mortality ratio/ or "years of potential life lost"/ OR survival analysis/ OR survival/ OR life expectancy/ OR death/ or "cause of death"/ | | | **1810217** | |
| 4 | #1 And #2 AND #3 | | | **1239** | |

| **Table S1b.** Search strategies (searches performed between 18-24 June 2024) | | | | | |
| --- | --- | --- | --- | --- | --- |
| **PubMed (All Field Vocabulary)** | | | | | |
| # | | Searches | | | Results |
| 1  Population | | "Severe mental illness" [All Fields] OR "Serious mental illness" [All Fields] OR "SMI" [All Fields] OR Bipolar [All Fields] OR "Manic depression" [All Fields] OR Psychosis [All Fields] OR Psychoses [All Fields] OR Schizophrenia [All Fields] OR Schizoaffective [All Fields] | | | **14336** |
| 2  Exposure | | Infection [All Fields] OR Infectious [All Fields] OR "Communicable disease" [All Fields] OR Virus [All Fields] OR Viral [All Fields] OR Bacteria* [All Fields] OR Fungus [All Fields] OR Fungal [All Fields] OR Parasit* [All Fields] OR Pneumonia [All Fields] OR Influenza [All Fields] OR COVID-19 [All Fields] OR Tuberculosis [All Fields] OR Sepsis [All Fields] OR [All Fields] Septicaemia OR [All Fields] Septicemia [All Fields] OR Hepatitis [All Fields] OR HIV [All Fields] | | | **340424** |
| 3  Outcome | | Death [All Fields] OR Mortality [All Fields] OR Life expectancy [All Fields] OR Survival [All Fields] | | | **219581** |
| 4 | | #1 AND #2 AND #3 | | | **175** |
| Additional filters | | ‘Human only’ | | | **175** |
| Additional filters | | ‘English language only’ | | | **175** |
| **PubMed (Controlled Vocabulary ‘MeSH terms’)** | | | | | |
| # | | Searches | Results | | |
| 1  Population | | Schizophrenia and disorders with psychotic features [MeSH Terms] OR Bipolar disorders [MeSH Terms] OR Affective psychoses [MeSH Terms] OR Disorder, psychotic [MeSH Terms] | **4899** | | |
| 2  Exposure | | Infectious disease [MeSH Terms] OR Viral disease [MeSH Terms] OR Virus [MeSH Terms] OR Bacteria [MeSH Terms] OR Parasitic disease [MeSH Terms] OR Pneumonia [MeSH Terms] OR influenza virus [MeSH Terms] OR tuberculosis [MeSH Terms] OR sepsis [MeSH Terms] OR septicemia [MeSH Terms] OR hepatitis a virus [MeSH Terms] OR hepatitis b virus [MeSH Terms] OR hepatitis c virus [MeSH Terms] OR hiv infection [MeSH Terms] | **135177** | | |
| 3  Outcome | | Life expectancy [MeSH Terms] OR Mortality [MeSH Terms] OR Analysis, survival [MeSH Terms] | **11480** | | |
| 4 | | #1 And #2 AND #3 | **0** | | |
| Additional filters | | ‘Human only’ | **0** | | |
| Additional filters | | ‘English language only’ | **0** | | |
| **Web of Science (All Field Vocabulary ‘Topic’)**  Note: Web of Science Core Collection employs no controlled vocabulary | | | | | |
| # | | Searches | | | Results |
| 1  Population | | Topic: "Severe mental illness" OR Topic: Serious mental illness OR Topic: SMI OR Topic: Bipolar OR Topic: Manic depression OR Topic: Psychosis OR Topic: Psychoses OR Topic: Schizophrenia OR Topic: Schizoaffective | | | **15323** |
| 2  Exposure | | Topic: Infection OR Topic: Infectious OR Topic: Communicable disease OR Topic: Virus OR Topic: Viral OR Topic: Bacteria* OR Topic: Fungus OR Topic: Fungal OR Topic: Parasit* OR Topic: Pneumonia OR Topic: Influenza OR Topic: COVID-19 OR Topic: Tuberculosis OR Topic: Sepsis OR Topic: Septicaemia OR Topic: Septicemia OR Topic: Hepatitis OR Topic: HIV | | | **282682** |
| 3  Outcome | | Topic: Death OR Topic: Mortality OR Topic: "Life expectancy" OR Topic: Survival | | | **218430** |
| 4 | | #1 And #2 AND #3 | | | **158** |
| Additional filters | | ‘English language only’ | | | **158** |
| **PsycINFO (All Field Vocabulary)** | | | | | |
| # | | Searches | | | Results |
| 1  Population | | Severe mental illness OR Serious mental illness OR SMI OR Bipolar OR Manic depression OR Psychosis OR Psychoses OR Schizophrenia OR  Schizoaffective | | | **7310** |
| 2  Exposure | | Infection OR Infectious OR Communicable disease OR Virus OR Viral OR Bacteria OR Bacterial OR Fungus OR Fungal OR Parasite OR Parasitic OR Pneumonia OR Influenza OR COVID-19 OR Tuberculosis OR Sepsis OR Septicaemia OR Septicemia OR Hepatitis OR HIV | | | **19443** |
| 3  Outcome | | Death OR Mortality OR Life expectancy OR Survival | | | **9013** |
| 4 | | #1 And #2 And #3 | | | **47** |
| **PsycINFO (Controlled Vocabulary ‘Subject heading search’)** | | | | | |
| # | | Searches | | Results | |
| 1  Population | | Serious mental illness/ OR bipolar disorder/ or exp bipolar i disorder/ or exp bipolar ii disorder/ or exp mania/ or exp affective psychosis/ OR  psychosis/ or affective psychosis/ or brief psychotic disorder/ or capgras syndrome/ or chronic psychosis/ or delusional disorder/ or paranoid psychosis/ or reactive psychosis/ or schizophrenia/ or paranoid schizophrenia/ OR schizophrenia/ or schizoaffective disorder/ | | **5271** | |
| 2  Exposure | | infectious disorders/ or bacterial infections/ or parasitic infections/ or pneumonia/ or sepsis/ or sexually transmitted diseases/ or viral infections/ OR pneumonia/ or coronavirus/ or covid-19/ OR influenza/ or swine influenza/ OR tuberculosis OR sepsis/ OR hepatitis/ OR hiv/ or aids/ | | **14109** | |
| 3  Outcome | | "death and dying"/ or mortality rate/ or mortality risk/ OR life expectancy/ | | **2536** | |
| 4 | | #1 And #2 And #3 | | **12** | |
| **EMBASE (All Field Vocabulary)** | | | | | |
| # | | Searches | | Results | |
| 1  Population | | Severe mental illness.tw OR Serious mental illness.tw OR SMI.tw OR Bipolar.tw OR Manic depression.tw OR Psychosis.tw OR Psychoses.tw OR Schizophrenia.tw OR Schizoaffective.tw | | **29773** | |
| 2  Exposure | | Infection.tw OR Infectious.tw OR Communicable disease.tw OR Virus.tw OR Viral.tw OR Bacteria*.tw OR Fungus.tw OR Fungal.tw OR Parasit*.tw OR Pneumonia.tw OR Influenza.tw OR COVID-19.tw OR Tuberculosis.tw OR Sepsis.tw OR Septicaemia.tw OR Septicemia.tw OR Hepatitis.tw OR HIV.tw | | **479212** | |
| 3  Outcome | | Death.tw OR Mortality.tw OR Life expectancy.tw OR Survival.tw | | **439496** | |
| 4 | | #1 AND #2 AND #3 | | **648** | |
| **EMBASE (Structured Vocabulary)** | | | | | |
| # | Searches | | | Results | |
| 1  Population | bipolar disorder/ or mania/ or bipolar depression/ or bipolar i disorder/ or bipolar ii disorder/ or bipolar mania/ OR psychosis/ or affective psychosis/ or schizophrenia/ OR schizophrenia/ or treatment-resistant schizophrenia/ | | | **25956** | |
| 2  Exposure | infection/ or virus infection/ OR communicable disease/ OR pneumonia/ OR influenza/ or swine influenza/ OR coronavirus disease 2019/ OR tuberculosis/ OR sepsis/ or septicemia/ OR virus hepatitis/ or hepatitis a/ or hepatitis b/ or hepatitis c/ OR Human immunodeficiency virus/ | | | **370278** | |
| 3  Outcome | mortality/ or all-cause mortality/ or infection fatality ratio/ or mortality rate/ or premature mortality/ or standardized mortality ratio/ or "years of potential life lost"/ OR survival analysis/ OR survival/ OR life expectancy/ OR death/ or "cause of death"/ | | | **279363** | |
| 4 | #1 And #2 AND #3 | | | **409** | |

2. List of data items which were extracted from each paper included in the review

| **Table S2.** Data extraction items |
| --- |
| Author, Title, Journal, Year, Study location, Funding source  Data source, Study design, Length of follow-up  Study population, Overall sample size, Age range/limits, Age, Sex, Ethnicity, SMI type, SMI assessment, SMI sample size, Control/comparison group, Control group sample size, Infection mortality outcome, Infection mortality assessment, Covariates  Main results (narrative account), Statistical test used, Test statistic (result), Inclusion of lived experience in study  Newcastle Ottawa study quality rating, Newcastle Ottawa number of stars |

3. PRISMA flow diagram for updated search (June 2024)

**Figure S1.** PRISMA flow diagram for updated search (June 2024)

Records excluded (n=49):

Conference abstracts (n=2)

Included in original search (n=20)

Review/commentary/letter to editor (n=6)

Non-English language (n=1)

Incorrect definition of SMI (n=5)

Incorrect outcome (n=11)

Association between SMI and outcome not explicitly assessed (n=4)

Records excluded because of COVID-19

(n=6)

**Studies included in review**

**(n=2)**

Studies included in review

(n=8)

Records assessed for eligibility

(n=57)

Records sought for retrieval

(n=58)

Records not retrieved

(n=1)

Records excluded

(n=874)

Duplicate records removed

(n=522)

Records screened

(n=932)

References identified from:

Databases (n=1449)

*PubMed (n=175)*

*Web of Science (n=158)*

*PsycINFO (n=59)*

*EMBASE (n=1057)*

Hand searches (n=5)

Total identified (n=1454)

4. List of excluded studies

| **Table S3a.** List of excluded studies (N=234) | | | |
| --- | --- | --- | --- |
| **Authors** | **Year** | **Study title** | **Reason for exclusion** |
| Abiodun | 1988 | Mortality in a psychiatric population: a Nigerian psychiatric hospital experience | No control group |
| Abrams et al | 2023 | Risk of death in women hospitalized with a primary diagnosis of urinary tract infection | Conference abstract |
| Abulseoud et al | 2022 | Attenuated initial serum ferritin concentration in critically ill coronavirus disease 2019 geriatric patients with comorbid psychiatric conditions | Incorrect definition of SMI |
| Ajetunmobi et al | 2013 | Early death in those previously hospitalised for mental healthcare in Scotland: a nationwide cohort study | Death from infection not included as an outcome |
| Alstrom | 1942 | Mortality in mental hospitals with especial regard to tuberculosis | Book |
| Altamura et al | 2002 | Mortality and suicidal risk in schizophrenia | Full text unavailable |
| Arbelo et al | 2021 | Psychiatric clinical profiles and pharmacological interactions in covid-19 inpatients referred to a consultation liaison psychiatry unit: A cross-sectional study | Incorrect definition of SMI |
| Babiagian et al | 1969 | The mortality experience of a population with psychiatric illness | Full text unavailable |
| Barbosa et al | 2016 | Causes of Death in an Acute Psychiatric Inpatient Unit of a Portuguese General Hospital | Incorrect definition of SMI |
| Bartels et al | 2020 | COVID-19 Emergency Reforms in Massachusetts to Support Behavioral Health Care and Reduce Mortality of People With Serious Mental Illness | Review/commentary/letter to editor |
| Benezech et al | 2011 | Mental patients and death at Cadillac Psychiatric Hospital: A 30-year study (1923-1952) | Non-English language |
| Berardi et al | 2021 | Mortality in mental health patients of the Emilia-Romagna region of Italy: a registry-based study | Incorrect definition of SMI |
| Berardi et al | 2018 | Mortality Rates and Trends Among Bologna Community Mental Health Service Users A 13-Year Cohort Study | The impact of SMI on infectious disease mortality was not specifically assessed |
| Bertolini et al | 2023 | Risk of SARS-cov-2 infection, severe COVID-19 illness and COVID-19 mortality in people with pre-existing mental disorders: an umbrella review | Review/commentary/letter to editor |
| Beyer et al | 2008 | Overall health and medical comorbidities in schizophrenia and bipolar disorder | Conference abstract |
| Bitter et al | 2017 | Mortality and the relationship of somatic comorbidities to mortality in schizophrenia. A nationwide matched-cohort study | Death from infection not included as an outcome |
| Black et al | 1988 | Mortality in schizophrenia--the Iowa Record-Linkage Study: a comparison with general population mortality | Death from infection not included as an outcome |
| Black et al | 1985 | The Iowa record-linkage study. II. Excess mortality among patients with organic mental disorders | Incorrect definition of SMI |
| Bobes et al | 2008 | Spanish consensus on physical health in patients with bipolar disorder | Non-English language |
| Bodryzlova et al | 2022 | The First Wave of COVID-19 in Forensic Psychiatry: A Rapid Review Series | Review/commentary/letter to editor |
| Boyer et al | 2022 | Impact of the COVID-19 pandemic on non-COVID-19 hospital mortality in patients with schizophrenia: A nationwide population-based cohort study | Death from infection not included as an outcome |
| Bralet et al | 2000 | Mortality in schizophrenia: A 8-year follow-up study in 150 chronic schizophrenics | Full text unavailable |
| Burnand & Schneider | 1946 | Investigation into tuberculosis in the mental hospital of Cery | Full text unavailable |
| Casadebaig et al | 1999 | Mortality among schizophrenic patients | Full text unavailable |
| Casey et al | 2011 | Schizophrenia: Medical illness, mortality, and aging | Review/commentary/letter to editor |
| Castro et al | 2021 | Mood Disorders and Outcomes of COVID-19 Hospitalizations | Incorrect definition of SMI |
| Chander et al | 2010 | Is there earlier time to death among hiv infected individuals with severe mental illness? | Conference abstract |
| Chen et al | 2021 | Longer-term mortality following SARS-cov-2 infection in people with severe mental illness: Retrospective case-matched study | Death from infection not included as an outcome |
| Chen et al | 2021 | Risk factors for excess deaths during lockdown among older users of secondary care mental health services without confirmed COVID-19: A retrospective cohort study | Death from infection not included as an outcome |
| Chen et al | 2020 | The early impact of COVID-19 on mental health and community physical health services and their patients' mortality in Cambridgeshire and Peterborough, UK | Death from infection not included as an outcome |
| Chen et al | 2021 | Excess mortality and risk factors for mortality among patients with severe mental disorders receiving home care case management | Death from infection not included as an outcome |
| Chevens | 1931 | The correlation of cause of death with type of insanity | No control group |
| Chou et al | 2013 | The incidence and all-cause mortality of pneumonia in patients with schizophrenia: a nine-year follow-up study | Death from infection not included as an outcome |
| Chouhan et al | 2022 | A study to determine the impact of stress on mental health in psychiatric patients of various races | Journal no longer considered scientific |
| Chu et al | 1966 | A fifteen-year follow-up of 214 psychiatric cases (chinese) - ii u ng | Full text unavailable |
| Closson et al | 2019 | HIV, schizophrenia, and all-cause mortality: A population-based cohort study of individuals accessing universal medical care from 1998 to 2012 in British Columbia, Canada | Death from infection not included as an outcome |
| Corrao et al | 2022 | Factors associated with severe or fatal clinical manifestations of SARS-cov-2 infection after receiving the third dose of vaccine | Death from infection not included as an outcome |
| Correll et al | 2022 | Mortality in people with schizophrenia: a systematic review and meta-analysis of relative risk and aggravating or attenuating factors | Review/commentary/letter to editor |
| Costardi et al | 2022 | COVID-19 contamination and severity among patients with schizophrenia: Results from a specialized outpatient clinic survey | Review/commentary/letter to editor |
| Croxford et al | 2019 | Mortality and causes of death among HIV patients in London in 2017 | Conference abstract |
| Cutler et al | 1990 | Postoperative complications in patients with disabling psychiatric illnesses or intellectual handicaps. A case-controlled, retrospective analysis | Incorrect definition of SMI |
| Daumit et al | 2010 | Pattern of mortality in a sample of Maryland residents with severe mental illness | The impact of SMI on infectious disease mortality was not specifically assessed |
| Daumit et al | 2006 | Adverse events during medical and surgical hospitalizations for persons with schizophrenia | No control group |
| Daumit et al | 2021 | Outcomes after COVID-19 diagnosis for people with versus without serious mental illness | Conference abstract |
| De Hert et al | 2022 | COVID-19-Related Mortality Risk in People With Severe Mental Illness: A Systematic and Critical Review | Review/commentary/letter to editor |
| De Hert et al | 2011 | Review of evidence concerning co-morbidity of schizophrenia and physical illness | Conference abstract |
| De Picker et al | 2021 | Closing COVID-19 mortality, vaccination, and evidence gaps for those with severe mental illness | Review/commentary/letter to editor |
| De Rezende | 2020 | Impact of comorbidities on survival estimate for patients with mental disorders | Conference abstract |
| Delibas et al | 2021 | Clinical Characteristics, Comorbid Medical Diagnoses, and Causes of Death of Individuals with Severe Mental Illness Who Died During Follow-up in Community Mental Health Centers: A Multicenter, Retrospective Study | No control group |
| Delorenze et al | 2010 | Mortality after diagnosis of psychiatric disorders and co-occurring substance use disorders among HIV-infected patients | Incorrect definition of SMI |
| Demler et al | 2018 | Implications of infection and trends of antibiotic prescribing in hospitalized patients diagnosed with serious mental illness | Incorrect definition of SMI |
| Dickerson | 2015 | Clinical and serological predictors of mortality in an expanded cohort of individuals with schizophrenia | Conference abstract |
| Dickerson et al | 2007 | Toxoplasma gondii in individuals with schizophrenia: association with clinical and demographic factors and with mortality | Death from infection not included as an outcome |
| Dickerson et al | 2016 | Mortality in schizophrenia and bipolar disorder: Clinical and serological predictors | Death from infection not included as an outcome |
| Dickerson et al | 2014 | Mortality in schizophrenia: clinical and serological predictors | Death from infection not included as an outcome |
| Dickerson et al | 2013 | ID: 1516847 serological predictors of mortality in a prospective schizophrenia cohort | Conference abstract |
| Dobre et al | 2023 | Clinical features and outcomes of COVID-19 patients hospitalized for psychiatric disorders: a French multi-centered prospective observational study | The impact of SMI on infectious disease mortality was not specifically assessed |
| Dragioti et al | 2023 | Impact of mental disorders on clinical outcomes of physical diseases: an umbrella review assessing population attributable fraction and generalized impact fraction | Review/commentary/letter to editor |
| Dutta et al | 2012 | Mortality in first-contact psychosis patients in the U.K.: a cohort study | Incorrect definition of SMI |
| Dutta et al | 2010 | Determining the long-term risk of suicide and premature death following a first episode of psychosis: An incidence cohort approach | Conference abstract |
| Duzgun et al | 2022 | The effect of risk factors on the clinical course and treatment of older patients with coronavirus disease 2019 | Incorrect definition of SMI |
| Ebuenyi et al | 2018 | The impact of co-morbid severe mental illness and HIV upon mental and physical health and social outcomes: A systematic review | Review/commentary/letter to editor |
| Egede et al | 2023 | Relationship between mental health diagnoses and COVID-19 test positivity, hospitalization, and mortality in Southeast Wisconsin | Death from infection not included as an outcome |
| Ekinci | 2022 | Comment on "Increased in-hospital mortality from COVID-19 in patients with schizophrenia". Considering the prevalence and protective factors of COVID-19 in patients with schizophrenia | Death from infection not included as an outcome |
| Evans et al | 2005 | Mood disorders in the medically ill: scientific review and recommendations | Review/commentary/letter to editor |
| Ezzouine et al | 2016 | Causes of admission in medical intensive care of patients suffering from severe psychiatric disorders | Conference abstract |
| Fekadu et al | 2018 | Excess mortality in severe mental illness: 10-year population-based cohort study in rural Ethiopia | No control group |
| Fond et al | 2023 | Lessons from the coronavirus disease 2019 pandemic in schizophrenia: a review | Review/commentary/letter to editor |
| Fond et al | 2021 | Mortality in schizophrenia: Towards a new health scandal? COVID-19 and schizophrenia | Review/commentary/letter to editor |
| Fond et al | 2021 | Association between mental health disorders and mortality among patients with COVID-19 in 7 countries: A systematic review and meta-analysis | Review/commentary/letter to editor |
| Fond et al | 2021 | Disparities in intensive care unit admission and mortality among patients with schizophrenia and COVID-19: A national cohort study | Death from infection not included as an outcome |
| Fond et al | 2023 | Mortality among inpatients with bipolar disorders and covid-19: A propensity score matching analysis in a national french cohort study | Death from infection not included as an outcome |
| Fond et al | 2021 | Increased in-hospital mortality from COVID-19 in patients with schizophrenia | Death from infection not included as an outcome |
| Fonseca et al | 2020 | Schizophrenia and COVID-19: risks and recommendations | Review/commentary/letter to editor |
| Franke et al | 2008 | Risk factors and mortality associated with default from multidrug-resistant tuberculosis treatment | Incorrect definition of SMI |
| Gale et al | 2021 | COVID-19 deaths in a secondary mental health service | No control group |
| Galea et al | 2021 | Mental health and mortality in a time of COVID-19 | Review/commentary/letter to editor |
| Garcia-Ribera et al | 2021 | Covid-19, hypercoagulability and risk of mortality in schizophrenia | Review/commentary/letter to editor |
| Gazdag et al | 2022 | Inpatient psychiatric care of COVID-19 infected patients in a Hungarian general hospital | Conference abstract |
| Ge et al | 2021 | Association of pre-existing comorbidities with mortality and disease severity among 167,500 individuals with COVID-19 in Canada: A population-based cohort study | Incorrect definition of SMI |
| Gholipour et al | 2021 | Statistical analysis of the Hungarian COVID-19 victims | No control group |
| Girardi et al | 2021 | Causes of mortality in a large population-based cohort of psychiatric patients in Southern Europe. | Incorrect definition of SMI |
| Goff et al | 2005 | Medical morbidity and mortality in schizophrenia: guidelines for psychiatrists | Full text unavailable |
| Griffith et al | 2017 | Identifying nursing home patients at risk of functional decline or death complicating hospitalization for pneumonia | Conference abstract |
| Grigoletti et al | 2009 | Mortality and cause of death among psychiatric patients: a 20-year case-register study in an area with a community-based system of care | Incorrect definition of SMI |
| Haas et al | 2020 | Mortality in people living with HIV and mental health disorders in South Africa | Conference abstract |
| Hassan et al | 2022 | COVID-19 infection, hospitalisation and mortality rates in people with severe mental illness: findings from two UK cohort studies | Conference abstract |
| Haugland et al | 1983 | Mortality in the era of deinstitutionalization | Incorrect definition of SMI |
| Havaki-Kontaxaki et al | 1995 | Mortality of patients in a large psychiatric hospital: II Causes of death and mortality of specific diagnostic categories of patients | Full text unavailable |
| Hayes et al | 2015 | A systematic review and meta-analysis of premature mortality in bipolar affective disorder | Review/commentary/letter to editor |
| Healy et al | 2012 | Mortality in schizophrenia and related psychoses: data from two cohorts, 1875-1924 and 1994-2010 | Death from infection not included as an outcome |
| Heila et al | 2005 | Mortality among patients with schizophrenia and reduced psychiatric hospital care | Death from infection not included as an outcome |
| Herrman et al | 1983 | A record-linkage study of mortality and general hospital discharge in patients diagnosed as schizophrenic | No statistical assessment performed |
| Hewer et al | 1996 | Mortality risk of psychiatric patients in inpatient acute treatment | Full text unavailable |
| Hewer et al | 1995 | Mortality among patients in psychiatric hospitals in Germany | Incorrect definition of SMI |
| Hoang et al | 2014 | Mortality following hospital discharge with a diagnosis of eating disorder: National record linkage study, England, 2001-2009 | Death from infection not included as an outcome |
| Hollister et al | 1960 | Causes of death in hospitalized veterans with neuropsychiatric disorders | Full text unavailable |
| Hussar | 1966 | Leading causes of death in institutionalized chronic schizophrenic patients: a study of 1,275 autopsy protocols | Full text unavailable |
| Hussar | 1966 | Leading causes of death in institutionalized chronic schizophrenic patients. A study of 1,275 autopsy protocols | Full text unavailable |
| Ifteni et al | 2014 | Sudden unexpected death in schizophrenia: autopsy findings in psychiatric inpatients | No control group |
| Ishida et al | 2019 | Mortality from sepsis among patients with schizophrenia and mood disorders in an intensive care unit: A chart review | Incorrect definition of SMI |
| Ishigami | 2021 | Risk Factors for Severe COVID-19 in a Large Medical Records Linkage System in the United States | Review/commentary/letter to editor |
| Ishii et al | 2018 | The limited impact of psychiatric disease and psychotropic medication on the outcome of hospitalization for pneumonia | Incorrect definition of SMI |
| J Robert | 1951 | Tuberculosis and dementia praecox | Full text unavailable |
| Jegede et al | 2021 | Clinical characteristics, hospital course, and outcomes among COVID-19 positive patients with mental illness in a community hospital in New York City | No control group |
| Jeon et al | 2021 | Association of mental disorders with SARS-cov-2 infection and severe health outcomes: Nationwide cohort study | Death from infection not included as an outcome |
| Johanson | 1958 | A study of schizophrenia in the male. A psychiatric and social study based on 138 cases with follow up | Book |
| Jones et al | 2020 | Associations of substance use, psychosis, and mortality among people living in precarious housing or homelessness: A longitudinal, community-based study in Vancouver, Canada | No control group |
| Jones et al | 2015 | Mortality from treatable illnesses in marginally housed adults: a prospective cohort study | Death from infection not included as an outcome |
| Kaiboriboon et al | 2013 | Long-term mortality in poor health and low income Americans with epilepsy | Conference abstract |
| Karaoulanis et al | 2021 | Do patients with schizophrenia have higher infection and mortality rates due to COVID-19? A systematic review | Review/commentary/letter to editor |
| Katz et al | 1957 | Tuberculosis in schizophrenia as compared with other types of mental disease | No control group |
| Khan et al | 2016 | Reducing morbidity and mortality from common medical conditions in schizophrenia: You play a vital role in systematic screening, initiating treatment, and maintaining follow-up | Review/commentary/letter to editor |
| Khaykin | 2009 | Prevention in the severely mentally ill: Primary care quality and adverse events among persons with schizophrenia, and the benefit of physical activity on sleep in a community sample of persons with severe mental illness | Full text unavailable |
| Kidd | 2008 | Respiratory tuberculosis in a mental hospital | Incorrect definition of SMI |
| Kim et al | 2022 | Effect of Comorbidities on the Infection Rate and Severity of COVID-19: Nationwide Cohort Study With Propensity Score Matching | No control group |
| Kiviniemi et al | 2010 | Regional differences in five-year mortality after a first episode of schizophrenia in Finland | Death from infection not included as an outcome |
| Koyama et al | 2022 | Mental Health Conditions and Severe COVID-19 Outcomes after Hospitalization, United States | Death from infection not included as an outcome |
| Kozloff et al | 2020 | The COVID-19 Global Pandemic: Implications for People With Schizophrenia and Related Disorders | Review/commentary/letter to editor |
| Kredenster et al | 2014 | . Cause and rate of death in people with schizophrenia across the lifespan: a population-based study in Manitoba, Canada. | Death from infection not included as an outcome |
| Kugathasan et al | 2019 | Increased mortality from somatic multimorbidity in patients with schizophrenia: a Danish nationwide cohort study | Death from infection not included as an outcome |
| Kuo et al | 2013 | Incidence and outcome of newly-diagnosed tuberculosis in schizophrenics: a 12-year, nationwide, retrospective longitudinal study | Death from infection not included as an outcome |
| Laursen et al | 2011 | Life expectancy among persons with schizophrenia or bipolar affective disorder | Death from infection not included as an outcome |
| Lawrence et al | 2013 | The gap in life expectancy from preventable physical illness in psychiatric patients in Western Australia: Retrospective analysis of population based registers | No statistical assessment performed |
| Lee et al | 2023 | COVID-19 vaccination, incidence, and mortality rates among individuals with mental disorders in South Korea: A nationwide retrospective study | Death from infection not included as an outcome |
| Lee et al. | 2023 | The nationwide trends in hospital admissions, deaths, and costs related to hepatitis C stratified by psychiatric disorders and substance use: an analysis of US hospitals between 2016 and 2019 | Death from infection not included as an outcome |
| Lee et al | 2020 | Risk of Mortality in Elderly Coronavirus Disease 2019 Patients With Mental Health Disorders: A Nationwide Retrospective Study in South Korea | Incorrect definition of SMI |
| Lee et al | 2021 | The outcomes of the COVID-19 infection among patients with psychiatric diagnosis | Conference abstract |
| Lee et al | 2021 | Clinical outcomes in COVID-19 patients with severe mental illness: A comparison between community-acquired and outbreak at sanatorium | Conference abstract |
| Lee et al | 2020 | Association between mental illness and COVID-19 susceptibility and clinical outcomes in South Korea: A nationwide cohort study | Death from infection not included as an outcome |
| Lega et al | 2021 | Psychiatric disorders among hospitalized patients deceased with COVID-19 in Italy | The impact of SMI on infectious disease mortality was not specifically assessed |
| Li et al | 2018 | Excess incidence and risk factors for recurrent pneumonia in bipolar disorder | No control group |
| Liao et al | 2013 | Cardiac complications associated with short-term mortality in schizophrenia patients hospitalized for pneumonia: a nationwide case-control study | No control group |
| Lindelius & Kay | 1973 | Some changes in the pattern of mortality in schizophrenia, in Sweden | No statistical assessment performed |
| Liu et al | 2021 | Mental and neurological disorders and risk of COVID-19 susceptibility, illness severity and mortality: A systematic review, meta-analysis and call for action | Review/commentary/letter to editor |
| Liu et al | 2017 | Excess mortality in persons with severe mental disorders: a multilevel intervention framework and priorities for clinical practice, policy and research agendas | Review/commentary/letter to editor |
| Loganathan et al | 2022 | Incidence and outcomes of COVID-19 first wave pandemic in a French nursing home with residents suffering from severe mental illnesses | Incorrect definition of SMI |
| Luciano et al | 2022 | Editorial: Mortality of people with severe mental illness: Causes and ways of its reduction | Review/commentary/letter to editor |
| Malomo et al | 2003 | Ten-year mortality review in a pioneer psychiatric hospital in West Africa | No control group |
| Manuelidis | 1952 | General morbidity in endogenous psychoses. Comparative statistics, especially of the incidence of tb, carcinoma and arteriosclerosis in sane and mentally diseased individuals | Full text unavailable |
| Marinho et al | 2020 | Psychosis among HIV-infected patients -a serious and complex association | Conference abstract |
| Mavreas et al | 2022 | COVID-19 pandemic and the mental health care system | Review/commentary/letter to editor |
| Mcguire et al | 2021 | Pneumonia | Full text unavailable |
| Melo et al | 2022 | All-cause and cause-specific mortality among people with severe mental illness in Brazil's public health system, 2000-15: A retrospective study | Incorrect definition of SMI |
| Miller et al | 2006 | Mortality and medical comorbidity among patients with serious mental illness | Incorrect definition of SMI |
| Miyashita et al | 2022 | Clinical features of nursing and healthcare-associated pneumonia due to COVID-19 | Incorrect definition of SMI |
| Mortensen et al | 1993 | Mortality and causes of death in first admitted schizophrenic patients | Death from infection not included as an outcome |
| Nab et al | 2023 | Changes in COVID-19-related mortality across key demographic and clinical subgroups: an observational cohort study using the opensafely platform on 18 million adults in England | Incorrect definition of SMI |
| Nab et al | 2023 | Changes in COVID-19-related mortality across key demographic and clinical subgroups in England from 2020 to 2022: a retrospective cohort study using the opensafely platform | Duplicate |
| Nadubinszky et al | 2022 | Influence of psychiatric diseases and psychiatric medication to the severity of clinical outcome of COVID-19 | Full text unavailable |
| Ndosi | 1997 | Causes of death among mental patients at Muhimbili Medical Centre, Dar es Salaam | Full text unavailable |
| Newman & Bland | 1991 | Mortality in a cohort of patients with schizophrenia: a record linkage study | Full text unavailable |
| Nguyen | 1982 | Fatal outcomes in schizophrenia (analysis of random hospital material for different forms of the disease) | Full text unavailable |
| Nielsen et al | 1977 | Follow-up 15 years after a geronto-psychiatric prevalence study: Conditions concerning death, cause of death, and life expectancy in relation to psychiatric diagnosis | Incorrect definition of SMI |
| Nielsen et al | 2013 | Increasing mortality gap for patients diagnosed with schizophrenia over the last three decades - A Danish nationwide study from 1980 to 2010 | Death from infection not included as an outcome |
| Nilsson | 1995 | Mortality in recurrent mood disorders during periods on and off lithium. A complete population study in 362 patients | Full text unavailable |
| Nilsson et al | 2022 | Adverse SARS-cov-2-associated outcomes among people experiencing social marginalisation and psychiatric vulnerability: A population-based cohort study among 4,4 million people | Incorrect definition of SMI |
| Nogueira et al | 2022 | Multimorbidity Profile of COVID-19 Deaths in Portugal during 2020 | No control group |
| Nourchene | 2022 | Schizophrenia in Covid-19 crisis : Is it a mortality risk factor ? | Conference abstract |
| Oakley et ak | 2018 | Increased mortality among people with schizophrenia and other non-affective psychotic disorders in the community: A systematic review and meta-analysis | Review/commentary/letter to editor |
| Odegard | 1936 | Mortality in Norwegian mental hospitals from 1916 to 1933 | Incorrect definition of SMI |
| Odegard | 1967 | Mortality in Norwegian psychiatric hospitals 1950-1962 | No statistical assessment performed |
| Oliveria et al | 2006 | Adverse events among nursing home residents with Alzheimer's disease and psychosis | Incorrect definition of SMI |
| Osman et al | 2020 | Mortality characteristics in Sudan in national psychiatric hospitals: 5-year review of hospital mortality | Incorrect definition of SMI |
| Pabis et al | 2021 | The impact of the COVID-19 pandemic on the schizophrenia: A literature review | Full text unavailable |
| Pardamean et al | 2022 | Mortality from coronavirus disease 2019 (Covid-19) in patients with schizophrenia: A systematic review, meta-analysis and meta-regression | Review/commentary/letter to editor |
| Peritogiannis et al | 2022 | Mortality in Schizophrenia-Spectrum Disorders: Recent Advances in Understanding and Management | Review/commentary/letter to editor |
| Petrova et al | 2022 | Outcomes of COVID-19 in Patients with Mental Disorders | Conference abstract |
| Piatt et al | 2010 | An examination of premature mortality among decedents with serious mental illness and those in the general population | Incorrect definition of SMI |
| Potapov et al | 2021 | Psychiatric complications of COVID-19 pneumonia: preliminary results of a single-centre prospective study | Conference abstract |
| Pranboon et al | 2020 | Prevalence of hospitalized in patients with epilepsy: A national data report from Thailand | Full text unavailable |
| Prince et al | 2007 | No health without mental health | Review/commentary/letter to editor |
| Rivas-Ramirez | 2021 | Patients with schizophrenia have decreased COVID-19 prevalence among hospitalised patients with psychiatric and neurological diseases: A retrospective analysis in Mexican population | No control group |
| Roe | 2022 | The link between Toxoplasma gondii infections and higher mortality in COVID-19 patients having schizophrenia | Review/commentary/letter to editor |
| Ruffieux et al | 2022 | Excess life-years lost associated with hospitalization for mental illness | Conference abstract |
| Ruiz et al | 2008 | Consensus on physical health of patients with schizophrenia from the Spanish Societies of Psychiatry and Biological Psychiatry | Full text unavailable |
| Saha et al | 2007 | A Systematic Review of Mortality in Schizophrenia | Review/commentary/letter to editor |
| Sartorius | 2007 | Physical illness in people with mental disorders | Review/commentary/letter to editor |
| Sato | 2019 | Comparison of cause of death in inpatients with schizophrenia and organic mental disorders: A survey of 20 years in a psychiatric hospital | Conference abstract |
| Schneider et al | 2019 | Mortality and Medical Comorbidity in the Severely Mentally Ill | Death from infection not included as an outcome |
| Schoepf et al | 2014 | Bipolar disorder and comorbidity: increased prevalence and increased relevance of comorbidity for hospital-based mortality during a 12.5-year observation period in general hospital admissions | No control group |
| Schoepf et al | 2016 | Medical comorbidity related risk factors for hospital-based mortality in psychiatric disorders of ICD-10 classes F1-F4: A comparative overview of five studies in general hospital admissions | Conference abstract |
| Schoepf et al | 2020 | Type-2 diabetes mellitus in schizophrenia: Increased prevalence and major risk factor of excess mortality in a naturalistic 7-year follow-up | No control group |
| Schoepf et al | 2014 | Physical comorbidity and its relevance on mortality in schizophrenia: a naturalistic 12-year follow-up in general hospital admissions | No control group |
| Schor et al | 2022 | A call to action: Increased mortality from COVID-19 among individuals with schizophrenia calls for coordinated vaccination efforts | Review/commentary/letter to editor |
| Schulz | 1933 | Mortality in general and mortality from tuberculosis in families of mental patients and in the average of the population | Full text unavailable |
| Servan Rendon-Luna | 2012 | Physical health problems of patients with severe mental illness | Conference abstract |
| Shafti et al | 2020 | Clinical Profile of Mortality among Chronic Schizophrenic Patients: A Local Pilot Survey in Iran | Full text unavailable |
| Shekhar et al | 2023 | Schizophrenia and COVID-19: A bibliometric analysis of trends and themes | Review/commentary/letter to editor |
| Shen et al | 2011 | Increased risks of acute organ dysfunction and mortality in intensive care unit patients with schizophrenia: A nationwide population-based study | Death from infection not included as an outcome |
| Shinn et al | 2020 | Perspectives on the COVID-19 Pandemic and Individuals With Serious Mental Illness | Review/commentary/letter to editor |
| Sikjaer et al | 2018 | The influence of psychiatric disorders on the course of lung cancer, chronic obstructive pulmonary disease and tuberculosis | Incorrect definition of SMI |
| Smith et al | 2022 | The Impact of COVID-19 on Psychiatric Services and Individuals with Serious Mental Illness in New York State during 2020 | Conference abstract |
| Soubani et al | 2023 | Septic shock short-term outcomes in hospitalized patients with major psychiatric disorders: Analysis from the national inpatient sample database | Death from infection not included as an outcome |
| Stroup et al | 2013 | Management of medical illness in persons with schizophrenia | Review/commentary/letter to editor |
| Suetani et al | 2021 | Increased rates of respiratory disease in schizophrenia: A systematic review and meta-analysis including 619,214 individuals with schizophrenia and 52,159,551 controls | Review/commentary/letter to editor |
| Suokas et al | 2022 | Mortality in persons with recent primary or secondary care contacts for mental disorders in Finland | Death from infection not included as an outcome |
| Susser et al | 2006 | HIV infection among young adults with psychotic disorders | No control group |
| Swaraj et al | 2019 | Meta-analysis of natural, unnatural and cause-specific mortality rates following discharge from in-patient psychiatric facilities | Review/commentary/letter to editor |
| Tabbane et al | 1993 | Mortality and causes of death in schizophrenia: A review | Full text unavailable |
| Tan et al | 2021 | Comparison of mental-physical comorbidity, risk of death and mortality among patients with mental disorders - A retrospective cohort study | Death from infection not included as an outcome |
| Teferra et al | 2011 | Five-year mortality in a cohort of people with schizophrenia in Ethiopia | Death from infection not included as an outcome |
| Termorshuizen et al | 2013 | Cause-Specific Mortality Among Patients With Psychosis: Disentangling the Effects of Age and Illness Duration | Death from infection not included as an outcome |
| Tokuda et al | 2008 | Acute care hospital mortality of schizophrenic patients | Death from infection not included as an outcome |
| Toubasi et al | 2021 | A meta-analysis: The mortality and severity of COVID-19 among patients with mental disorders | Review/commentary/letter to editor |
| Tsai et al | 2005 | A retrospective analysis of risk and protective factors for natural death in bipolar disorder | Full text unavailable |
| Tsuang et al | 1980 | Premature deaths in schizophrenia and affective disorders. An analysis of survival curves and variables affecting the shortened survival | Death from infection not included as an outcome |
| Turki et al | 2022 | Bidirectional Associations Between Covid-19 Infection And Mental Disorders | Conference abstract |
| Tweed et al | 2022 | Premature mortality in people affected by co-occurring homelessness, justice involvement, opioid dependence, and psychosis: a retrospective cohort study using linked administrative data | Death from infection not included as an outcome |
| Tweed et al | 2019 | The health of people experiencing multiple forms of social exclusion: a systematic review | Conference abstract |
| Tweed et al | 2021 | Health of people experiencing co-occurring homelessness, imprisonment, substance use, sex work and/or severe mental illness in high-income countries: a systematic review and meta-analysis | Review/commentary/letter to editor |
| UK GOV | 2023 | Premature mortality in adults with severe mental illness (SMI) | Review/commentary/letter to editor |
| UK GOV | 2018 | Severe mental illness (SMI) and physical health inequalities: briefing | Review/commentary/letter to editor |
| Usui & Kaneko | 1973 | Annual changes in the number of deaths caused by mental diseases and the mortality rate per prefecture (Japanese) | Full text unavailable |
| Vai et al | 2021 | Mental disorders and risk of COVID-19-related mortality, hospitalisation, and intensive care unit admission: a systematic review and meta-analysis | Review/commentary/letter to editor |
| Vai et al | 2022 | Joint European policy on the COVID-19 risks for people with mental disorders: An umbrella review and evidence- and consensus-based recommendations for mental and public health | Review/commentary/letter to editor |
| Valent | 2021 | Age, comorbidities, nursing home stay and outcomes of SARS-cov-2 infection in a Northern Italian cohort | Incorrect definition of SMI |
| Vieweg et al | 1995 | Medical disorders in the schizophrenic patient | Review/commentary/letter to editor |
| Vita et al | 2022 | The impact of the Covid-19 pandemic on patients with schizophrenia | Review/commentary/letter to editor |
| Vrotsou et al | 2021 | Variables associated with COVID-19 severity: an observational study of non-paediatric confirmed cases from the general population of the Basque Country, Spain | Incorrect definition of SMI |
| Vrotsou et al | 2021 | Factors determining the COVID-19 infection outcome in people 65 years of age | Conference abstract |
| Wan et al | 2020 | Prognosis analysis of patients with mental disorders with COVID-19: a single-center retrospective study | Incorrect definition of SMI |
| Wang et al | 2021 | Increased risk of COVID-19 infection and mortality in people with mental disorders: Analysis from electronic health records in the United States | The impact of SMI on infectious disease mortality was not specifically assessed |
| Watkins et al | 1972 | Causes of death in psychiatric hospitals | Full text unavailable |
| Weisberg et al | 2016 | Mortality after critical care hospitalization in patients with psychotic disorders | Conference abstract |
| Weiser et al | 2021 | Infection, Mortality and Vaccinations for COVID-19 in Patients With Schizophrenia and Bipolar Disorder: Data From an Entire Population | Conference abstract |
| Werbeloff et al | 2014 | Premature death is higher in persons with psychotic disorders but not with psychotic experiences: A population based longitudinal study | Conference abstract |
| Westermeyer | 1978 | Mortality and psychosis in a peasant society | Incorrect definition of SMI |
| WHO | 2017 | Helping people with severe mental disorders live longer and healthier lives: policy brief | Review/commentary/letter to editor |
| WHO | 2018 | Management of physical health conditions in adults with severe mental disorders | Review/commentary/letter to editor |
| Wolf et al | 2016 | Infection in people with severe mental illness | Review/commentary/letter to editor |
| Wood et al | 1985 | Mortality variations among public mental health patients | The impact of SMI on infectious disease mortality was not specifically assessed |
| Yang et al | 2021 | Should people with severe mental illness be prioritized for the COVID-19 vaccination? | Review/commentary/letter to editor |
| Zilber et al | 1985 | Mortality among psychiatric patients – the groups at risk. | Death from infection not included as an outcome |

| **Table S3b.** List of studies excluded as outcome was COVID-19 mortality (N=30) | | |
| --- | --- | --- |
| **Authors** | **Year** | **Study title** |
| Barcella et al | 2021 | Severe mental illness is associated with increased mortality and severe course of covid-19 |
| Beaney et al | 2022 | Trends and associated factors for Covid-19 hospitalisation and fatality risk in 2.3 million adults in England |
| Bowersox et al | 2023 | COVID-19 mortality among veterans with serious mental illness in the veterans health administration |
| Catalan et al | 2023 | Psychosis and substance abuse increase the COVID-19 mortality risk |
| Crapanzano et al | 2022 | Factors influencing elevated mortality rates of patients with schizophrenia hospitalized with covid |
| Das-Munshi et al | 2021 | All-cause and cause-specific mortality in people with mental disorders and intellectual disabilities, before and during the COVID-19 pandemic: cohort study |
| Descamps et al | 2022 | Association between mental disorders and COVID-19 outcomes among inpatients in France: A retrospective nationwide population-based study |
| Goldberger et al | 2022 | COVID-19 and severe mental illness in Israel: testing, infection, hospitalization, mortality and vaccination rates in a countrywide study |
| Hassan et al | 2022 | Disparities in COVID-19 infection, hospitalisation and death in people with schizophrenia, bipolar disorder, and major depressive disorder: a cohort study of the UK Biobank |
| Hassan et al | 2023 | Heightened COVID-19 Mortality in People With Severe Mental Illness Persists After Vaccination: A Cohort Study of Greater Manchester Residents |
| Hippisley-Cox et al | 2023 | QCovid 4 - Predicting risk of death or hospitalisation from COVID-19 in adults testing positive for SARS-CoV-2 infection during the Omicron wave in England |
| Maripuu et al | 2021 | Death Associated With Coronavirus (COVID-19) Infection in Individuals With Severe Mental Disorders in Sweden During the Early Months of the Outbreak-An Exploratory Cross-Sectional Analysis of a Population-Based Register Study |
| Moga et al | 2021 | Inflammatory Response in SARS-CoV-2 Infection of Patients with Schizophrenia and Long-Term Antipsychotic Treatment |
| Murphy et al | 2022 | Hospitalization, Mechanical Ventilation, and Mortality After COVID-19 Among Adults With or Without Serious Mental Illness |
| Nemani et al | 2021 | Association of Psychiatric Disorders With Mortality Among Patients With COVID-19 |
| Padhi et al | 2022 | Association of Severe Acute Respiratory Syndrome Coronavirus 2 Infection and Related Mortality Rates with Mental Disorders: An Epidemiological Correlation in the Indian Population |
| Rodriguez-Molinero et al | 2020 | Association between COVID-19 prognosis and disease presentation, comorbidities and chronic treatment of hospitalized patients |
| Sanchez-Rico et al | 2022 | Is a Diagnosis of Schizophrenia Spectrum Disorder Associated With Increased Mortality in Patients With COVID-19? |
| Schultebraucks et al | 2023 | The impact of preexisting psychiatric disorders and antidepressant use on COVID-19 related outcomes: a multicenter study |
| Schwarzinger et al | 2023 | Mental disorders, COVID-19-related life-saving measures and mortality in France: A nationwide cohort study |
| Seon et al | 2021 | Risk of COVID-19 diagnosis and death in patients with mental illness: A cohort study |
| Teixeira et al | 2021 | Analysis of COVID-19 Infection and Mortality Among Patients With Psychiatric Disorders, 2020 |
| Tokuda et al | 2023 | Serious mental illness and in-hospital mortality among hospitalized patients with acute COVID-19: A large-database analysis in Japan |
| Tyson et al | 2021 | Predictors of survival in older adults hospitalized with COVID-19 |
| Tzur-Bitan et al | 2021 | COVID-19 hospitalisation, mortality, vaccination, and postvaccination trends among people with schizophrenia in Israel: a longitudinal cohort study |
| Tzur-Bitan et al | 2021 | COVID-19 Prevalence and Mortality Among Schizophrenia Patients: A Large-Scale Retrospective Cohort Study |
| Tzur-Bitan et al | 2022 | Severe breakthrough COVID-19 infections in vaccinated patients with schizophrenia in Israel |
| Wallin et al | 2022 | Risk of severe COVID-19 infection in individuals with severe mental disorders, substance use disorders, and common mental disorders |
| Xu et al | 2023 | Excess deaths from COVID-19 among Medicare beneficiaries with psychiatric diagnoses: Community versus nursing home |
| Yang et al | 2020 | Pre-pandemic psychiatric disorders and risk of COVID-19: a UK Biobank cohort analysis |

| **Table S3c.** List of excluded studies from updated search in 2024 (N=50) | | | |
| --- | --- | --- | --- |
| **Authors** | **Year** | **Study title** | **Reason for exclusion** |
| Abrams et al | 2023 | Risk of death in women hospitalized with a primary diagnosis of urinary tract infection | Conference abstract |
| Aslam et al | 2023 | Treatment outcomes and adverse drug reactions among patients with drug-resistant tuberculosis receiving all-oral, long-term regimens: First record viewing report from Pakistan | The impact of SMI on infectious disease mortality was not specifically assessed |
| Bertolini et al | 2023 | Risk of SARS-CoV-2 infection, severe COVID-19 illness and COVID-19 mortality in people with pre-existing mental disorders: an umbrella review | Included in original screen |
| Biazus et al | 2023 | All-cause and cause-specific mortality among people with bipolar disorder: a large-scale systematic review and meta-analysis | Review/commentary/letter to editor |
| Bodryzlova et al | 2023 | The first wave of COVID-19 in forensic psychiatry: A rapid review series | Included in original screen |
| Bowersox et al | 2023 | COVID-19 mortality among veterans with serious mental illness in the veteran’s health administration | Included in original screen |
| Bransfield et al | 2023 | Microbes and Mental Illness: Past, Present, and Future | Review/commentary/letter to editor |
| Castro et al | 2024 | Underlying Mental Illness and Risk of Severe Outcomes Associated With COVID-19 | Incorrect definition of SMI |
| Catalan et al | 2023 | Psychosis and substance abuse increase the COVID-19 mortality risk | Included in original screen |
| Cheng et al | 2023 | Risk of All-Cause and Suicide Death in Patients With Schizophrenia: An Entire-Population Longitudinal Study in Taiwan | Death from infection not included as an outcome |
| Copeland et al | 2023 | A case-control study of antipsychotic use and pneumonia-related mortality in the United Kingdom | Incorrect definition of SMI |
| D’Andrea et al | 2023 | Exposure to psychotropic medications and COVID-19 course after hospital admission: Results from a prospective cohort study | Included in original screen |
| Das-Munshi et al | 2023 | Severe mental illness, race/ethnicity, multimorbidity and mortality following COVID-19 infection: Nationally representative cohort study | Death from infection not included as an outcome |
| Dragioti et al | 2023 | Impact of mental disorders on clinical outcomes of physical diseases: an umbrella review assessing population attributable fraction and generalized impact fraction | Review/commentary/letter to editor |
| Dutta et al | 2007 | Suicide and other causes of mortality in bipolar disorder: a longitudinal study | Death from infection not included as an outcome |
| Egede et al | 2023 | Relationship between mental health diagnoses and COVID-19 test positivity, hospitalization, and mortality in Southeast Wisconsin | Included in original screen |
| Fond et al | 2023 | Lessons from the coronavirus disease 2019 pandemic in schizophrenia: A review | Included in original screen |
| Fond et al | 2023 | Mortality among inpatients with bipolar disorders and COVID-19: A propensity score matching analysis in a national French cohort study | Included in original screen |
| Getahun et al | 2023 | Survival status and risk factors for mortality among multidrug-resistant tuberculosis patients in Addis Ababa, Ethiopia: A retrospective follow-up study | Incorrect definition of SMI |
| Hassan et al | 2023 | Heightened COVID-19 Mortality in People with Severe Mental Illness Persists After Vaccination: A Cohort Study of Greater Manchester Residents | Included in original screen |
| Hoertel et al | 2024 | Excess mortality and its causes among older adults with schizophrenia versus those with bipolar disorder and major depressive disorder: a 5-year prospective multicenter study | Death from infection not included as an outcome |
| István et al | 2023 | Psychiatric disorders are associated with high mortality rates: somatic comorbidity and mortality in autism spectrum disorder and schizophrenia | Non-English language |
| Kowalski et al | 2023 | Schizophrenia and the COVID-19 pandemic: A narrative review from the biomedical perspective | Review/commentary/letter to editor |
| Lakbar et al | 2023 | Association of severe mental illness and septic shock case fatality rate in patients admitted to the intensive care unit: A national population-based cohort study | Included in original screen |
| Lakbar et al | 2024 | Severe mental illness and mortality in sepsis and septic shock: a systematic review and meta-analysis | Review/commentary/letter to editor |
| Lee et al | 2023 | The nationwide trends in hospital admissions, deaths, and costs related to hepatitis C stratified by psychiatric disorders and substance use: an analysis of US hospitals between 2016 and 2019 | Included in original screen |
| Lee et al | 2023 | COVID-19 vaccination, incidence, and mortality rates among individuals with mental disorders in South Korea: A nationwide retrospective study | Included in original screen |
| Molero et al | 2023 | COVID-19 risk, course and outcome in people with mental disorders: a systematic review and meta-analyses | Review/commentary/letter to editor |
| Moreno-Juste et al | 2024 | Mental health and risk of death and hospitalization in COVID-19 patients. Results from a large-scale population-based study in Spain | Death from infection not included as an outcome |
| Mpango et al | 2023 | Physical and psychiatric comorbidities among patients with severe mental illness as seen in Uganda | Death from infection not included as an outcome |
| Nab et al | 2023 | Changes in COVID-19-related mortality across key demographic and clinical subgroups in England from 2020 to 2022: a retrospective cohort study using the OpenSAFELY platform | Included in original screen |
| Ng et al | 2023 | Decreasing trend of inpatient mortality rates of aseptic versus septic revision total hip arthroplasty: an analysis of 681,034 cases | The impact of SMI on infectious disease mortality was not specifically assessed |
| Ni et al | 2023 | Discharge Against Medical Advice After Hospitalization for Sepsis: Predictors, 30-Day Readmissions, and Outcomes | The impact of SMI on infectious disease mortality was not specifically assessed |
| Olaya et al | 2023 | Association between mental disorders and mortality: A register-based cohort study from the region of Catalonia | Included in original screen |
| Olivieri-Mui et al | 2023 | Health care utilization and mortality at the intersection of frailty and morbidity among older Medicare beneficiaries with HIV | Unavailable – not published |
| Ranger et al | 2023 | Preexisting Neuropsychiatric Conditions and Associated Risk of Severe COVID-19 Infection and Other Acute Respiratory Infections | Included in original screen |
| Ruffieux et al | 2023 | Life years lost associated with mental illness: A cohort study of beneficiaries of a South African medical insurance scheme | Death from infection not included as an outcome |
| Schultebraucks et al | 2023 | The impact of preexisting psychiatric disorders and antidepressant use on COVID-19 related outcomes: a multicenter study | Included in original screen |
| Schwarzinger et al | 2023 | Mental disorders, COVID-19-related life-saving measures and mortality in France: A nationwide cohort study | Included in original screen |
| Soubani et al | 2023 | Septic Shock Short-Term Outcomes in Patients With Psychiatric Disorders: Analysis From the National Inpatient Sample Database | Included in original screen |
| Stenberg et al | 2023 | The effects of sociodemographic factors and comorbidities on sepsis: A nationwide Swedish cohort study | Death from infection not included as an outcome |
| Tokuda et al | 2023 | Serious mental illness and in-hospital mortality among hospitalized patients with acute COVID-19: A large-database analysis in Japan | Included in original screen |
| Trickey et al | 2024 | Longitudinal trends in causes of death among adults with HIV on antiretroviral therapy in Europe and North America from 1996 to 2020: a collaboration of cohort studies | Incorrect definition of SMI |
| Tsai et al | 2024 | Performance of the Elixhauser Comorbidity Index in Predicting Mortality Among a National US Sample of Hospitalized Homeless Adults | Death from infection not included as an outcome |
| Tzur Bitan | 2023 | Severe COVID-19 and breakthrough infections in vaccinated schizophrenia patients: A matched controlled cohort study | Conference abstract |
| Xie et al | 2024 | Long-term outcomes following hospital admission for COVID-19 versus seasonal influenza: a cohort study | Death from infection not included as an outcome |
| Xu et al | 2023 | Excess deaths from COVID-19 among Medicare beneficiaries with psychiatric diagnoses: Community versus nursing home | Included in original screen |
| Xu et al | 2024 | Clinical Characteristics and Fatality Risk Factors for Patients with Listeria monocytogenes Infection: A 12-Year Hospital-Based Study in Xi'an, China | The impact of SMI on infectious disease mortality was not specifically assessed |
| Yang et al | 2024 | Causal associations between severe mental illness and sepsis: a Mendelian randomization study | Incorrect definition of SMI |
| Ye et al | 2024 | COVID-19 pandemic amplified mortality rates among adolescents with bipolar disorder through family-related factors | Death from infection not included as an outcome |

| **Table S3d.** List of studies excluded as outcome was COVID-19 mortality (updated search June 2024, N=6) | | |
| --- | --- | --- |
| **Authors** | **Year** | **Study title** |
| Cheng et al | 2023 | Risk factors for poor COVID-19 outcomes in patients with psychiatric disorders |
| De Oliviera et al | 2024 | The association between schizophrenia and increased COVID-19 mortality in a cohort of over 2 million people in Brazil |
| Delgado et al | 2023 | Investigational medications in 9,638 hospitalized patients with severe COVID-19: lessons from the "fail-and-learn" strategy during the first two waves of the pandemic in 2020 |
| Gibbs et al | 2024 | COVID-19-associated mortality in individuals with serious mental disorders in Sweden during the first two years of the pandemic- a population-based register study |
| Lopez-Cuadrado et al | 2023 | Clinical characteristics and outcomes of people with severe mental disorders hospitalized due to COVID-19: A nationwide population-based study |
| Yiu et al | 2023 | Risks of COVID-19-related hospitalisation and mortality among individuals with mental disorders following BNT162b2 and CoronaVac vaccinations: A case-control study |

5. Study quality assessment

Study quality was assessed using the Newcastle-Ottawa Scale. AR and MH agreed on the following criteria for assessing study quality in this area:

- In studies that looked at general populations (e.g. the general population of a city or country), the SMI sample should be representative of people with SMI in the population. Therefore, studies that assessed SMI using hospital inpatient registers only were not awarded a star for the ‘representativeness of exposed cohort’ category. This is because a small proportion of people with severe mental health disorders received inpatient care – approximately 1.8% in England^1^. To be considered representative SMI assessment will need to come from at least two sources (e.g. both inpatient registry and primary care record). Studies that looked at the impact of SMI on mortality within specific groups of patients with infection (e.g. people hospitalised with sepsis) were considered to have representative samples.
- All studies were awarded no stars for the ‘Demonstration that outcome of interest was not present at start of study’ category. In the case of mortality studies, the outcome of interest is considered to be the presence of a disease/incident rather than death, i.e. a statement of no history of disease earns a star. In most cases of infectious disease, the possibility that a person had a history of the infection of interest (e.g. influenza, sepsis) cannot definitively be ruled out and as such, no paper included a statement of this kind.
- For the ‘Follow-up long enough for outcomes to occur’ category, 12 months was considered to be an adequate follow-up period for studies that looked at general populations. This is due to the acute nature of the development and course of infections. In studies that looked at the impact of SMI on mortality within specific groups of patients with infection (e.g. people hospitalised with sepsis), any follow-up period was deemed acceptable

| **Table S4.** Quality assessment of included studies (N=27) | | | | | | | | | | |
| --- | --- | --- | --- | --- | --- | --- | --- | --- | --- | --- |
|  | **Representativeness of exposed cohort** (max 1 star) | **Selection of non-exposed cohort** (max 1 star) | **Ascertainment of exposure** (max 1 star) | **Demonstration that outcome of interest was not present at start of study**  (max 1 star) | **Comparability of cohorts based on design/analysis**  (max 2 stars) | **Assessment of outcome** (max 1 star) | **Follow-up long enough for outcomes to occur** (max 1 star) | **Adequacy of follow-up of the cohort** (max 1 star) | **Total quality score** | **Quality rating** |
| Alleback & Wistedt, 1986 | 0 | 1 | 1 | 0 | 1 | 1 | 1 | 1 | **6** | **Fair** |
| Almeida et al. 2016 | 1 | 1 | 1 | 0 | 1 | 1 | 1 | 1 | **7** | **Good** |
| Brown et al. 2010 | 1 | 1 | 1 | 0 | 1 | 1 | 1 | 1 | **7** | **Good** |
| Buda et al. 1988 | 0 | 1 | 1 | 0 | 1 | 1 | 1 | 0 | **5** | **Fair** |
| Castagnini et al. 2013 | 1 | 1 | 1 | 0 | 1 | 1 | 1 | 1 | **7** | **Good** |
| Chan et al. 2021 | 1 | 1 | 1 | 0 | 1 | 1 | 1 | 1 | **7** | **Good** |
| Chen et al. 2011 | 1 | 1 | 1 | 0 | 2 | 1 | 1 | 1 | **8** | **Good** |
| Cheng et al. 2014 | 0 | 1 | 1 | 0 | 1 | 1 | 1 | 1 | **6** | **Fair** |
| Crump et al. 2013  (bipolar disorder) | 1 | 1 | 1 | 0 | 2 | 1 | 1 | 1 | **8** | **Good** |
| Crump et al. 2013 (schizophrenia) | 1 | 1 | 1 | 0 | 2 | 1 | 1 | 1 | **8** | **Good** |
| Hiroeh et al. 2008 | 0 | 1 | 1 | 0 | 1 | 1 | 1 | 1 | **6** | **Fair** |
| John et al. 2018 | 1 | 1 | 1 | 0 | 1 | 1 | 1 | 1 | **7** | **Good** |
| Kendler et al. 1986 | 0 | 1 | 1 | 0 | 1 | 1 | 1 | 1 | **6** | **Fair** |
| Ko et al. 2018 | 0 | 1 | 1 | 0 | 1 | 1 | 1 | 1 | **6** | **Fair** |
| Lakbar et al. 2023 | 1 | 1 | 1 | 0 | 2 | 1 | 1 | 1 | **8** | **Good** |
| Lesage et al. 2015 | 1 | 1 | 1 | 0 | 1 | 1 | 1 | 1 | **7** | **Good** |
| Mortensen et al. 1990 | 0 | 1 | 1 | 0 | 1 | 1 | 1 | 1 | **6** | **Fair** |
| Nilsson et al. 2021 | 1 | 1 | 1 | 0 | 0 | 1 | 1 | 1 | **6** | **Poor** |
| Olaya et al. 2023 | 1 | 1 | 1 | 0 | 2 | 1 | 1 | 1 | **8** | **Good** |
| Olfson et al. 2015 | 1 | 1 | 1 | 0 | 2 | 1 | 1 | 1 | **8** | **Good** |
| Osby et al. 2000 | 0 | 1 | 1 | 0 | 1 | 1 | 1 | 1 | **6** | **Fair** |
| Osby et al. 2001 | 0 | 1 | 1 | 0 | 1 | 1 | 1 | 1 | **6** | **Fair** |
| Oud & Garza. 2022 | 1 | 1 | 1 | 0 | 2 | 1 | 1 | 1 | **8** | **Good** |
| Ranger et al. 2023 | 1 | 1 | 1 | 0 | 2 | 1 | 1 | 1 | **8** | **Good** |
| Ribe et al. 2015 | 1 | 1 | 1 | 0 | 1 | 1 | 1 | 1 | **7** | **Good** |
| Talaslahti et al. 2012 | 0 | 1 | 1 | 0 | 1 | 1 | 1 | 1 | **6** | **Fair** |
| Tsuang et al. 1980 | 0 | 1 | 0 | 0 | 1 | 0 | 1 | 1 | **4** | **Poor** |
| Yazdani et al. 2022 | 1 | 1 | 1 | 0 | 2 | 1 | 1 | 1 | **8** | **Good** |
| Yung et al. 2020 | 1 | 1 | 1 | 0 | 1 | 1 | 1 | 1 | **7** | **Good** |

6. Leave-one-out analyses

**Figure S2.** Leave-one-out analysis for Infectious disease overall. This indicated that Talaslahti et al’s study may have been unduly contributing to the overall pooled effect size seeing as it reduced considerably when this study was removed. Therefore, the decision was made to perform the meta-analysis without this study.

**Figure S4.** Leave-one-out analysis for Sepsis.

**Figure S3.** Leave-one-out analysis for Respiratory infections.

**Figure S5.** Leave-one-out analysis for Other infections.

7. Subgroup analyses: Infectious disease overall

Subgroup analyses for each meta-analyses are tabulated below. Any significant differences that emerged between subgroups are displayed graphically in forest plots.

| **Table S5.** Subgroup analyses for Infectious disease overall | | | | |
| --- | --- | --- | --- | --- |
| **Potential source of heterogeneity and p value** | **Subgroup** | **Number of analyses** | **Pooled RR (95% CI)** | **I^2^** |
| SMI type (p=0.579) | SMI | 1 | - | - |
|  | Schizophrenia and other psychoses | 18 | 2.69 (2.19 to 3.31) | 74.0% |
|  | Bipolar disorder | 5 | 3.06 (2.33 to 4.02) | 16.6% |
| **Gender (p=0.044)** | All | 14 | 2.61 (2.14 to 3.18) | 75.3% |
|  | Men | 5 | 3.55 (2.58 to 4.89) | 46.8% |
|  | Women | 5 | 2.14 (1.70 to 2.71) | 0.0% |
| Study quality (p=0.547) | Good | 10 | 2.90 (2.52 to 3.34) | 43.6% |
|  | Fair/poor | 14 | 2.62 (1.95 to 3.52) | 76.5% |
| Type of control group (p=0.570) | Population | 22 | 2.76 (2.31 to 3.29) | 70.0% |
|  | Cohort/patient | 2 | 2.42 (1.60 to 3.66) | 21.3% |

**Figure S5.** Subgroup analyses examining gender as a source of heterogeneity for the association between SMI and risk of death from Infectious disease overall.

8. Funnel plot: Infectious disease overall

**Figure S6.** Funnel plot for Infectious diseases overall meta-analysis. The Egger’s test indicates that no publication bias was present (p=0.936)

9. Subgroup analyses: Respiratory infections

Subgroup analyses for each meta-analyses are tabulated below. Any significant differences that emerged between subgroups are displayed graphically in forest plots.

| **Table S6.** Subgroup analyses for Respiratory infections | | | | |
| --- | --- | --- | --- | --- |
| **Potential source of heterogeneity and p value** | **Subgroup** | **Number of analyses** | **Pooled RR (95% CI)** | **I^2^** |
| SMI type (p=0.742) | SMI | 3 | 2.81 (1.89 to 4.16) | 96.8% |
|  | Schizophrenia and other psychoses | 10 | 3.39 (2.40 to 4.80) | 99.3% |
|  | Bipolar disorder | 5 | 3.41 (2.06 to 5.64) | 96.7% |
| **Type of respiratory infection (p=0.051)** | Pneumonia | 7 | 4.15 (3.42 to 5.04) | 90.8% |
|  | Influenza/pneumonia/SARI | 11 | 2.78 (1.95 to 3.96) | 99.3% |
| Gender (p=0.433) | All | 14 | 3.55 (2.75 to 4.60) | 99.0% |
|  | Men | 2 | 2.46 (1.10 to 5.54) | 91.4% |
|  | Women | 2 | 244 (1.29 to 4.62) | 86.7% |
| Study quality (p=0.803) | Good | 15 | 3.24 (2.50 to 4.21) | 99.1 |
|  | Fair/poor | 3 | 3.64 (1.52 to 8.73) | 90.7 |
| **Type of control group (p=0.024)** | Population | 11 | 3.87 (2.76 to 5.42) | 99.1 |
|  | Cohort/patient | 7 | 2.53 (2.19 to 2.93) | 88.7 |

**Figure S7.** Subgroup analyses examining type of respiratory infection as a source of heterogeneity for the association between SMI and risk of death from respiratory infections

**Figure S8.** Subgroup analyses examining type of control group as a source of heterogeneity for the association between SMI and risk of death from respiratory infections

10. Funnel plot: Respiratory infections

**Figure S9.** Funnel plot for Respiratory infection meta-analysis. The Egger’s test indicates that no publication bias was present (p=0.389)

11. Subgroup analyses: Sepsis

| **Table S7.** Subgroup analyses for Sepsis | | | | |
| --- | --- | --- | --- | --- |
| **Potential source of heterogeneity and p value** | **Subgroup** | **Number of analyses** | **Pooled RR (95% CI)** | **I^2^** |
| **SMI type (p<0.001)** | SMI | 3 | 2.27 (1.50 to 3.43) | 86.0% |
|  | Schizophrenia and other psychoses | 3 | 1.34 (0.37 to 4.88) | 99.9% |
|  | Bipolar disorder | 1 | - | - |
| Study quality (p=0.901) | Good | 6 | 1.52 (0.65 to 3.56) | 99.8% |
|  | Fair/poor | 1 | - | - |
| **Type of control group (p=0.006)** | Population | 3 | 2.81 (1.29 to 6.16) | 98.7% |
|  | Patient | 4 | 0.90 (0.72 to 1.13) | 95.5% |

**Figure S10.** Subgroup analyses examining SMI type as a source of heterogeneity for the association between SMI and risk of death from Sepsis

**Figure S11.** Subgroup analyses examining control type as a source of heterogeneity for the association between SMI and risk of death from Sepsis

12. Funnel plot: Sepsis mortality

**Figure S12.** Funnel plot for Sepsis mortality meta-analysis of log-transformed standardised mortality ratios. The Egger’s test indicates that no publication bias is present (p=0.789)

13. Subgroup analyses: Other Infections

| **Table S8.** Subgroup analyses for Other infections | | | | |
| --- | --- | --- | --- | --- |
| **Potential source of heterogeneity and p value** | **Subgroup** | **Number of analyses** | **Pooled RR (95% CI)** | **I^2^** |
| **SMI type (p<0.032)** | SMI | 6 | 2.20 (1.73 to 2.81) | 0.0% |
|  | Schizophrenia and other psychoses | 2 | 1.16 (0.68 to 1.98) | 75.5% |
| Study quality (p=0.249) | Good | 7 | 1.90 (1.33 to 2.73) | 62.8% |
|  | Fair/poor | 1 | - | - |
| Type of control group (p=0.333) | Population | 2 | 1.52 (1.21 to 1.91) | 0.0% |
|  | Patient/cohort | 6 | 1.94 (1.26 to 2.98) | 69.0% |

**Figure S13.** Subgroup analyses examining SMI type as a source of heterogeneity for the association between SMI and risk of death from Other infections

14. Funnel plot: Other infection mortality

**Figure S14.** Funnel plot for other infection mortality meta-analysis of log-transformed standardised mortality ratios. The Egger’s test indicates that publication bias is present (p=0.201)

15. Meta-regressions for all outcomes

| **Table S9.** Metaregressions assessing the impact of continuous variables on the pooled effect size for each infection mortality outcome | | | | | | | | |
| --- | --- | --- | --- | --- | --- | --- | --- | --- |
|  | Infectious disease overall | | Respiratory infections | | Sepsis | | Other infections | |
|  | β | P value | β | P value | β | P value | β | P value |
| SMI sample size | 1.02 | 0.056 | 0.98 | 0.253 | **0.97** | **0.049** | 0.97 | 0.072 |
| Number of covariates | 0.99 | 0.931 | 0.93 | 0.273 | **0.88** | **0.013** | **0.89** | **0.029** |
| Follow-up period | **0.87** | **0.003** | 1.00 | 0.991 | 1.03 | 0.338 | 1.04 | 0.488 |

Supplementary Materials: References

1 Degli Esposti M, Ziauddeen H, Bowes L, *et al.* Trends in inpatient care for psychiatric disorders in NHS hospitals across England, 1998/99–2019/20: an observational time series analysis. *Soc Psychiatry Psychiatr Epidemiol* 2022; **57**: 993–1006.
